# Priming with HDAC Inhibitors Sensitizes Ovarian Cancer Cells to Treatment with Cisplatin and HSP90 Inhibitors

**DOI:** 10.3390/ijms21218300

**Published:** 2020-11-05

**Authors:** Ana J. Rodrigues Moita, Jan J. Bandolik, Finn K. Hansen, Thomas Kurz, Alexandra Hamacher, Matthias U. Kassack

**Affiliations:** 1 Institute for Pharmaceutical and Medicinal Chemistry, University of Duesseldorf, 40225 Duesseldorf, Germany; ana.rodrigues.moita@hhu.de (A.J.R.M.); jan.bandolik@hhu.de (J.J.B.); thomas.kurz@hhu.de (T.K.); alexandra.hamacher@hhu.de (A.H.); 2 Pharmaceutical Institute, University of Bonn, 53121 Bonn, Germany; finn.hansen@uni-bonn.de

**Keywords:** ovarian cancer, chemoresistance, HSP90 inhibitors, luminespib, HSP990, epigenetics, HDAC inhibitors, panobinostat, LMK235, cisplatin

## Abstract

Ovarian cancer is the fifth leading cause of cancer deaths. Chemoresistance, particularly against platinum compounds, contributes to a poor prognosis. Histone deacetylase inhibitors (HDACi) and heat shock protein 90 inhibitors (HSP90i) are known to modulate pathways involved in chemoresistance. This study investigated the effects of HDACi (panobinostat, LMK235) and HSP90i (luminespib, HSP990) on the potency of cisplatin in ovarian cancer cell lines (A2780, CaOV3, OVCAR3 and cisplatin-resistant sub-clones). Preincubation with HDACi increased the cytotoxic potency of HSP90i, whereas preincubation with HSP90i had no effect. Preincubation with HSP90i or HDACi 48h prior to cisplatin enhanced the cisplatin potency significantly in all cell lines via apoptosis induction and affected the expression of apoptosis-relevant genes and proteins. For CaOV3CisR and A2780CisR, a preincubation with HDACi for 48–72 h led to complete reversal of cisplatin resistance. Furthermore, permanent presence of HDACi in sub-cytotoxic concentrations prevented the development of cisplatin resistance in A2780. However, triple combinations of HDACi, HSP90i and cisplatin were not superior to dual combinations. Overall, priming with HDACi sensitizes ovarian cancer cells to treatment with HSP90i or cisplatin and has an influence on the development of cisplatin resistance, both of which may contribute to an improved ovarian cancer treatment.

## 1. Introduction

Ovarian cancer is among the five most deadly types of cancer in women [1]. Although therapeutic options have improved in the last decades, the five-year survival rate remains at around 48% for ovarian cancer in the USA [2] and 40–43% in Germany [3,4]. When comparing the different cancers of the female genital tract, ovarian cancer shows the highest death rate (6.9 per 100,000 US or German citizen) compared to cervical cancer (2.3 per 100,000 in USA and 2.4 per 100,000 in Germany) and uterine cancer (4.8 per 100,000 in USA and 3.0 per 100,000 in Germany). In 2020, about 22,000 new cases of ovarian cancer are predicted in the USA with around 14,000 deaths [3,5]. First line therapy includes taxane- and platinum-based chemotherapy after cytoreductive surgery [6]. A major obstacle of this therapy is the development of resistance against platinum-based drugs eventually leading to death. Resistance is multifactorial and can be intrinsic or acquired [7]. In addition, there is the possibility of neoadjuvant chemotherapy (NACT) to further reduce the tumor mass before debulking surgery [8]. Randomized studies have shown that NACT before debulking was “non-inferior” to primary tumor removal [9,10]. However, there is currently no consensus on which patients are best suited for this type of therapy. Mutations and altered gene expression are the most common reasons for resistance, leading to altered mismatch repair, DNA methylation, histone acetylation and reduced apoptosis [11,12]. Modulators to overcome platinum resistance have not yet entered clinical routine, possibly due to multiple mutations in resistant cells and heterogeneous cell populations [13]. Heat shock protein 90 (HSP90) is an abundantly found molecular chaperone with over 280 client proteins and influences their activity through various regulatory mechanisms [14,15]. Many HSP90 client proteins such as Akt, MEK, receptor tyrosine kinases or estrogen receptors are members of proliferative and antiapoptotic pathways activated in drug-resistant ovarian cancers [16]. HSP90 inhibitors (HSP90i) could therefore interfere in many pathways by inducing degradation of HSP90 client proteins through the ubiquitin-proteasome pathway [17]. Moreover, the expression of HSP90 is increased in most cancer types [18]. For that reason, HSP90i are widely tested in different cancers such as colon, melanoma, prostate or ovarian cancer as single drug or as part of combination therapies [19,20,21,22,23,24]. In the past, HSP90i such as luminespib, SNX-5422, onalespib or ganetespib have entered phase II clinical trials (NLM identifier NCT01854034, NCT02612285, NCT2535338, and NCT01551693). Most of them are finished now. HSP90 was identified as a promising target in epithelial ovarian cancer [16]. Our group has previously shown that increased expression and phosphorylation of the HSP90 clients IGFR, ErbB2, and ErbB3 play a pivotal role in the development of resistance in ovarian cancer [25,26]. Thus, we investigated in this study whether HSP90i such as luminespib and HSP990 could serve as treatment options to address cisplatin resistance. 

Another widely tested approach to address chemoresistance is epigenetic modulation [27,28]. Specifically, histone deacetylase inhibitors (HDACi) are currently under investigation. HDACi increase the acetylation status of histones and non-histone proteins such as transcription factors or HSP90 [13,29]. Furthermore, HDACi influence cell proliferation, cell cycle regulation and apoptosis of ovarian cancer cells [30]. We and others demonstrated that HDACi, e.g., the broad spectrum HDACi vorinostat, panobinostat, and LMK235 or class I selective HDACi entinostat, sensitize ovarian cancer cells to DNA-damaging drugs such as cisplatin [31,32,33]. HDACi increase the acetylation of HSP90 and lead to a loss of chaperone activity [34,35]. Thus, the combination of HSP90i and HDACi seems to be intriguing for the treatment of cancer. The combination of HSP90i and HDACi has indeed been tested: a combination of the HSP90i luminespib and the HDACi belinostat increased cell death in anaplastic thyroid carcinoma cells in a synergistic manner [34]. Based on this study, the detected synergism was investigated further, and it was found that the HSP90i SNX-5422 is also synergistic in combination with belinostat, vorinostat, and trichostatin A [36]. There is further evidence for a synergistic cytotoxic effect of the combination panobinostat or vorinostat with luminespib or HSP990 [29,37]. Part of the effect was shown to be mediated through inactivation of the PI3K/AKT signaling pathway, which was previously shown by us to contribute to cisplatin resistance in ovarian cancer and triple negative breast cancer [25,38]. Similar effects were obtained by combining luminespib and the HDACi vorinostat in multiple myeloma cells [29]. To the best of our knowledge, the combination of HSP90i and HDACi has not yet been tested in ovarian cancer cells together with a cytotoxic platinum agent. Thus, we decided to include the HSP90i luminespib and HSP990, as well as the non-selective HDACi panobinostat and LMK235 [39] in our study and to investigate their effect in a combination therapy with cisplatin in ovarian cancer. The rationale of this project was to compare single and combined effects of the HDACi and HSP90i and cisplatin in the ovarian cancer cell lines A2780 and A2780CisR to contribute to improved therapies addressing chemoresistance in ovarian cancer. The best combination was further tested in CaOV3, OVCAR3, and their cisplatin-resistant sub-cell lines to extend the impact of our findings on high grade serous ovarian cancer cell lines.

## 2. Results

### 2.1. HDACi and HSP90i Mediated Biological Effects in Ovarian Cancer Cells

First, we characterized the HSP90i luminespib and HSP990 and the HDACi panobinostat and LMK235 for their biological effects in the ovarian cancer cell lines A2780 and A2780CisR. MTT assays were used to determine the compound-induced cytotoxicity and revealed IC_50_ values in the two-digit nanomolar range for both HSP90i and panobinostat. LMK235 showed three-digit nanomolar IC_50_ values against A2780 (847 nM) and A2780CisR (644 nM) (Table 1 and Figure S2A,B). 

**Figure 1:**
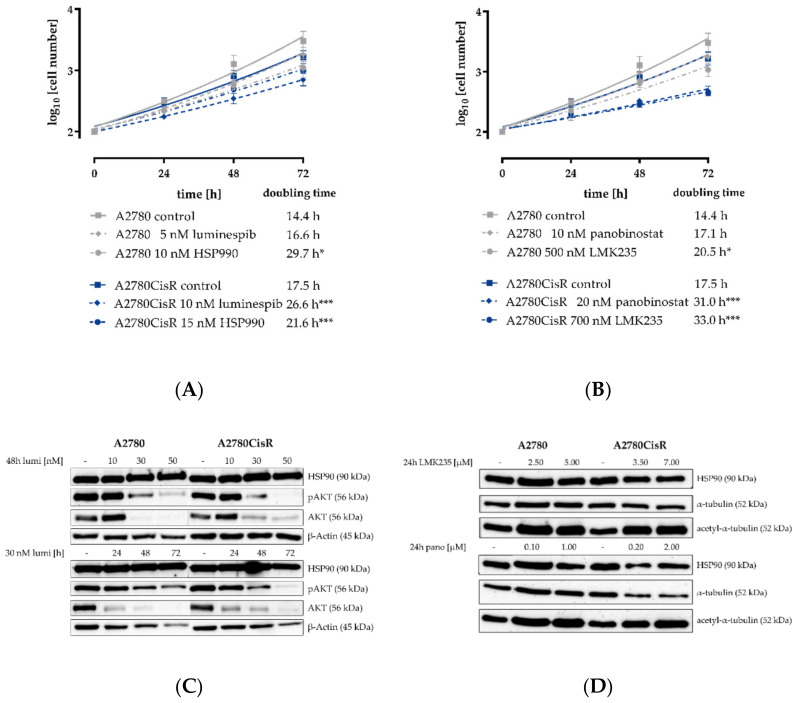
Effects of HSP90i and HDACi on cell growth and protein expression in A2780 and A2780CisR cells. (**A**) A 72 h incubation with the HSP90i luminespib and HSP990 showed a slight decrease in cell proliferation for A2780CisR cells. In A2780, this effect was only achieved with HSP990. (**B**) A 72 h incubation with the HDACi panobinostat or LMK235 slightly reduced the cellular growth of A2780 and A2780CisR cells. (**C**) A 48 h incubation with luminespib with IC_50_, three-fold IC_50_ and five-fold IC_50_ or time-dependent incubation with three-fold IC_50_ of luminespib led to a decrease in the expression of the HSP90 client protein AKT and its phosphorylation (pAKT). The uncropped and labeled immunoblots are shown in Figure S1. (**D**) A 24 h incubation with panobinostat or LMK235 increased the acetylation of α-tubulin in both cell lines. Data shown are mean ± SEM of three independent experiments or one representative immunoblot out of three. Statistical analysis was performed using t-test. Levels of significance: * *p* ≤ 0.05, *** *p* ≤ 0.001.

To analyze the influence of the observed cytotoxicity on the growth kinetics of A2780 and A2780CisR, we analyzed the doubling times with or without inhibitor treatment after 24, 48, and 72 h. The results are shown in Figure 1A,B. Luminespib and HSP990 led to a slight increase in the doubling time of A2780 and A2780CisR. This effect was statistically significant for all four compounds in A2780CisR but only for two compounds (HSP990 and LMK235) in A2780 (Figure 1A). Further, both HDACi reduced the growth of A2780 and A2780CisR, as indicated by an increase in the doubling time (Figure 1B). In accordance to HSP90i, the effect seemed stronger in A2780CisR. The effect of HSP90i and HDACi on the protein expression, protein phosphorylation (HSP90i) and protein acetylation was analyzed by Western blot. The results are shown in Figure 1C,D. Both HSP90i reduced the expression and phosphorylation of the client protein and proto-oncogene AKT in both cell lines in a concentration- and time-dependent manner (luminespib in Figure 1C and HSP990 in Figure S3). This is consistent with the mechanisms of HSP90 inhibition. Cellular activity of both HDACi was demonstrated by a concentration-dependent increase in acetylated tubulin in A2780 and A2780CisR analyzed by Western blot (Figure 1D).

### 2.2. Treatment Order of HDACi and HSP90i Affects Increase in Cytotoxic Activity and Apoptosis Induction in A2780 and A2780CisR

**Figure 2:**
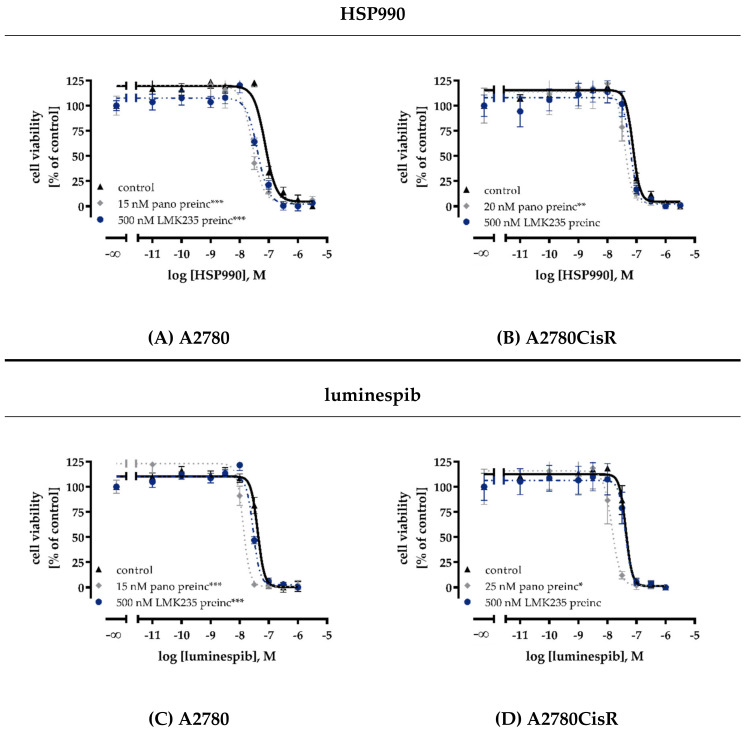
Preincubation with HDACi increase cytotoxic potency of HSP90i. A 48 h preincubation (preinc) with panobinostat or LMK235 with the indicated concentrations decreased IC_50_ values of HSP990 (**A**,**B**) and luminespib (**C**,**D**) in A2780 (**A**,**C**) and A2780CisR cells (**B**,**D**). IC_50_ values, pIC_50_ and SEM are shown in Table S1. Data shown are mean ± SEM of three independent experiments each carried out in triplicate. Statistical analysis was performed using t-test. Levels of significance: * *p* ≤ 0.05, ** *p* ≤ 0.01, *** *p* ≤ 0.001.

Because of the known interplay between HSP90 and HDACs [13,29,34,36,37], we were interested in analyzing the cytotoxic effects of a combination treatment with HDACi and HSP90i. MTT experiments for this combination study used longer incubation times than standard 72 h MTT assays. A 48 h preincubation with one inhibitor (HDACi or HSP90i) was followed by 72 h coincubation with HDACi or HSP90i resulting in a total incubation time of 120 h. Therefore, IC_50_ values for inhibitors obtained in this experimental setup were substantially higher (Table S1 and Figure 2A–D) and not comparable to those obtained with a 72 h MTT (Figure S2). A 48 h preincubation with HSP90i (luminespib or HSP990, respectively) had no effect on the cytotoxic activity of panobinostat or LMK235 (Figure S4). In contrast, 48 h preincubation with panobinostat or LMK235 increased the potency of HSP990 and luminespib by factors up to 3.2-fold (Figure 2A–D and Figure S4 and Table S1). The effects on the potency of HSP90i were significant in A2780 and for panobinostat in A2780CisR. For LMK235, no significant interaction in A2780CisR was observed. Therefore, we analyzed apoptosis induction only for the combination of panobinostat with both HSP90i. The results are shown in Figure S5. The enhancement of the HSP90i-induced cytotoxicity by HDACi pretreatment seen in MTT assays was mediated by apoptosis induction. A 24 h preincubation with HSP90i followed by 24 h coincubation with panobinostat had no effect on the number of apoptotic cells, whereas, vice versa, a 24 h preincubation with panobinostat followed by 24 h coincubation with HSP90i significantly increased the rate of apoptosis in A2780 and A2780CisR cells (Figure S5). 

**Figure 3:**
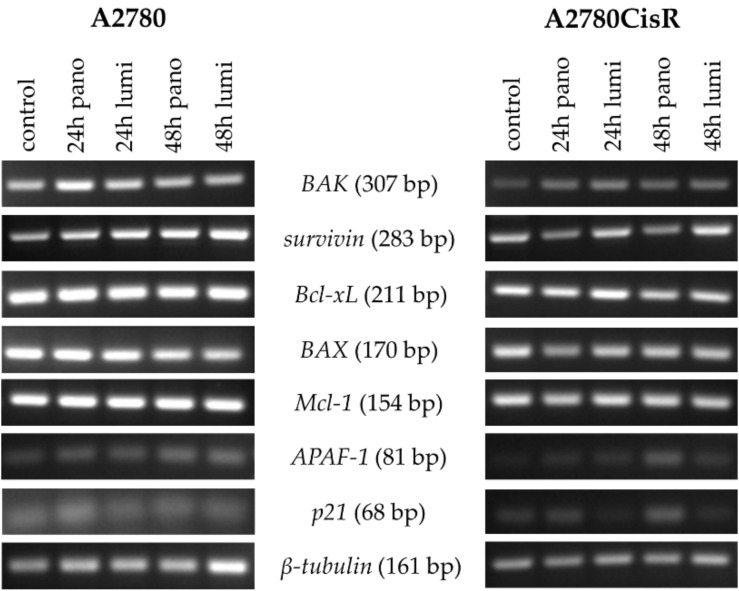
Effects of HDACi or HSP90i incubation on apoptosis-related genes. Gene expression data were obtained by PCR. Cells were treated with 10 nM (A2780) or 20 nM (A2780CisR) panobinostat or 5 nM (A2780) or 10 nM (A2780CisR) luminespib for 24 or 48 h.

To gain a first idea of the mechanisms behind the observed enhancement of cytotoxicity and apoptosis induction after preincubation with HDACi, the effects of panobinostat and luminespib on the gene expression of apoptosis-relevant factors in A2780 and A2780CisR cells were investigated. Both inhibitors (luminespib and panobinostat) were chosen based on their highest effects in the MTT assays shown in Figure 2A–D. Expression of the tumor suppressor gene *p21*; the proapoptotic genes *BAK*, *BAX,* and *APAF-1*; and the antiapoptotic genes *survivin*, *Bcl-xL,* and *Mcl-1* was analyzed by PCR. A2780 and A2780CisR cells were treated with panobinostat or luminespib alone for the indicated time points followed by analysis of gene expression. The results are shown in Figure 3.

In A2780, the expression of pro- and antiapoptotic genes remained largely unaffected by the treatment with the exception of *survivin*. Surprisingly, both inhibitors increased the mRNA level in a time-dependent manner. In A2780CisR, panobinostat reduced *survivin* expression and increased *APAF-1* and *p21* expression.

### 2.3. Effects of HDACi and HSP90i on Cisplatin Induced Cytotoxicity and Apoptosis

HDACi showed a priming effect on the cytotoxic activity of HSP90i. This prompted us to explore a possible influence of HSP90i or HDACi treatment alone or in combination on the potency of cisplatin in ovarian cancer cells. First, MTT assays were performed in A2780 and A2780CisR with a 48 h inhibitor preincubation prior to cisplatin administration or a coincubation of inhibitor and cisplatin (Figure S6A–D, IC_50_ values in Table 2 and pIC_50_ values in Table S2A). Coincubation of inhibitors with cisplatin (dual combination) showed no or only small increases in the potency of cisplatin in A2780/A2780CisR except for LMK235 (Figure S6C and Table 2). A 48 h preincubation with the inhibitors prior to cisplatin administration however markedly increased the potency of cisplatin with shift factors up to 6.7 (e.g., calculated for A2780, IC_50 (cisplatin)_: 3.34 µM; IC_50 (cisplatin + 48h HSP990 preincubation)_: 0.5 µM; Figure S6A and Table 2). Notably, the effects were more pronounced in A2780 cells. HDACi and HSP90i showed equal effects in increasing the cisplatin potency in A2780 (Figure S6A,C). 

In A2780CisR, HDACi were slightly but not significantly superior to HSP90i (Figure S6B,D). The best effect was observed with a 48 h preincubation with 20 nM panobinostat, decreasing the cisplatin IC_50_ from 19.7 to 6.53 µM (shift factor 3). Despite this relatively large effect, a complete resensitization by reaching the cisplatin IC_50_ of the parental cell line was not achieved (Figure S6D and Table 2). Notably, even though HDACi and HSP90i were equally effective in increasing cisplatin potency in MTT assays in A2780 (Figure S6A,C), only HDACi, but not HSP90i, showed a significant increase in apoptosis induction in the absence or presence of cisplatin (Figure S6E).

Preincubation with HDACi or HSP90i increased the potency of cisplatin. Next, we were interested in whether a triple combination of HDACi, HSP90i, and cisplatin had an even more pronounced effect regarding chemosensitivity against cisplatin. Data are presented in Figure S7A,B and Table 2. HSP90i were incubated for 48 h prior to addition of cisplatin plus HDACi (Table 2). In a further experiment, the HDACi were incubated for 48 h prior to addition of cisplatin plus HSP90i (Table 2). pIC_50_ values and errors are shown in Table S2. All triple combinations (HDACi panobinostat or LMK235 plus HSP90i luminespib or HSP990 plus cisplatin) resulted in significant increases in the potency of cisplatin in A2780 and A2780CisR (Figure S7A,B). The increase in potency yielded shift factors for triple combinations not significantly superior to those obtained by dual combinations (i.e., HDACi or HSP90i plus cisplatin). As an example, the triple combination consisting of a 48 h preincubation with panobinostat followed by 72 h of HSP990 plus cisplatin gave the lowest IC_50_ for cisplatin in A2780CisR (5.91 µM, Figure S7B). However, cisplatin potency was not significantly different from a dual combination of panobinostat with cisplatin which gave an IC_50_ of 6.53 µM (Figure S6D). Shift factors from dual (HDACi plus cisplatin) and triple combination MTT assays are listed in Table S3. 

The effect of inhibitors was more pronounced in A2780 than in A2780CisR indicated by shift factors up to 6.7 in A2780 and up to 3.3 in A2780CisR. We conclude that a triple combination has no benefit over a dual combination treatment with regard to the cytotoxicity of cisplatin.

**Figure 4:**
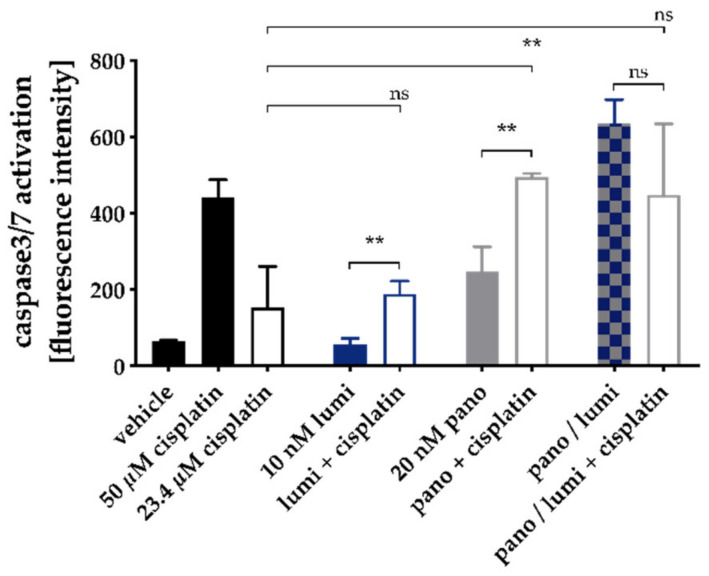
Caspase 3/7 activation by single treatment and combinations of panobinostat, luminespib, and cisplatin in A2780CisR cells. A2780CisR cells were preincubated with panobinostat and/or luminespib (lumi) for 48h. Cisplatin (23.4 µM, corresponding to the IC_50_) or buffer control were added for a further incubation period of 24 h. Caspase 3/7 activation was analyzed by ArrayScan XTI. Cisplatin 50 µM (24 h) served as positive control for caspase 3/7 activation, 0.2% DMSO as vehicle control. Data are the mean ± SD. Statistical analysis to compare the caspase 3/7 activation of the two indicated treatments was performed using t-test. Levels of significance: ns *p* > 0.05, ** *p* ≤ 0.01.

Similar to the dual combinations, the observed increase in cytotoxic activity of the triple combinations was mediated via apoptosis induction. A 48 h preincubation with HDACi in combination with HSP90i induced apoptosis in a similar range as a single pretreatment with HDACi (absence of cisplatin; Figures S6E and S7C). Further, pretreatment with HDACi plus HSP90i increased the cisplatin-mediated induction of apoptosis (Figure S7C). However, this effect was not superior to dual combinations of HDACi and cisplatin (Figure S6E). To confirm that the observed effects in the apoptosis assay were mediated via caspase activation, the activity of caspase 3 and 7 was measured with a fluorescence-based assay for single treatment and combinations of panobinostat, luminespib, and cisplatin. The results are shown in Figure 4.

Whereas 10 nM luminespib did not lead to caspase 3/7 activation, 20 nM panobinostat was as effective as the IC_50_ of cisplatin (23.4 µM). The combination of luminespib with cisplatin resulted in a caspase 3/7 activation not significantly different from cisplatin alone. The HSP90i luminespib did not increase cisplatin efficacy for caspase 3/7 activation, which is in agreement with the results from apoptosis activation (Figure S6E). However, preincubation with panobinostat significantly increased caspase 3/7 activation of cisplatin (Figure 4). In agreement with our results from MTT and apoptosis assays (Figures S6 and S7), the triple combination of HSP90i, HDACi, and cisplatin showed no advantages over dual combinations of HDACi and cisplatin. Again, in agreement with results from MTT and apoptosis induction (Figure 2 and Figure S5), the combination of panobinostat and luminespib led to a massive caspase 3/7 activation which could not further be increased by cisplatin. 

Notably, the antiproliferative and chemosensitizing effects of HSP90i and HDACi alone or in combination were not associated with changes in the cell cycle distribution of A2780 or A2780CisR (Figure S8). 

### 2.4. Effects of HDACi and/or HSP90i Plus Cisplatin Incubation on mRNA and Protein Expression of Pro-/Antiapoptotic Key Genes

**Figure 5:**
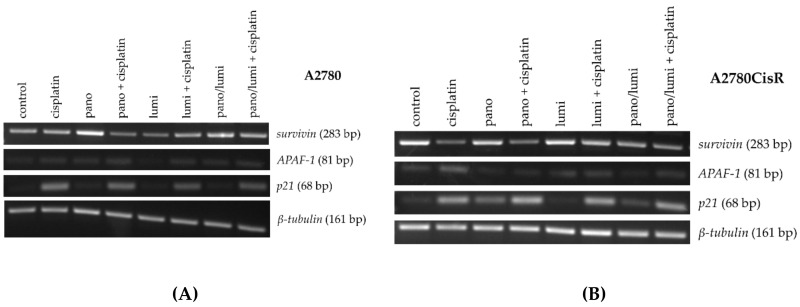
Effects of HDACi or HSP90i preincubation prior to cisplatin on apoptosis-related genes. Gene expression data were obtained by PCR. (**A**,**B**) A2780 and A2780CisR cells were treated with the indicated inhibitors or their combinations. For combination treatments panobinostat and/or luminespib were administrated 48 h prior to 24 h cisplatin treatment in an IC_50_. Cell line specific untreated controls were obtained by 72 h incubation with cell culture medium. The concentrations used (**A**,**B**) were the same as for cytotoxicity combination studies (Figure S6 and Table 2). One representative agarose gel is shown. Densitometric evaluation can be found in Figure S10B,C.

To identify genes that are altered by dual or triple combinations, we performed a PCR analysis. The tumor suppressor gene *p21*, the proapoptotic gene *APAF-1,* and antiapoptotic gene *survivin* were analyzed (Figure 5).

**Figure 6:**
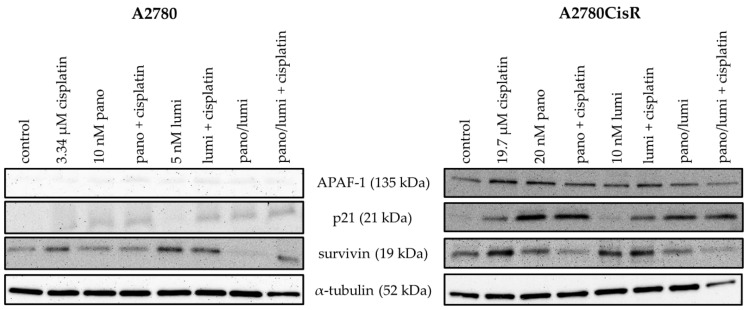
Effects of HDACi and HSP90i incubation or preincubation prior to cisplatin on protein expression levels of pro-/antiapoptotic proteins. Representative immunoblot analysis of APAF-1, *p21*, survivin and α-tubulin (as loading control) in A2780 and A2780CisR cells is shown. For combination treatments, panobinostat (pano) and/or luminespib (lumi) were administrated 48 h prior to 24 h cisplatin treatment with respective IC_50_ concentrations. Cell line specific control was treated with vehicle for 72 h. Densitometric analysis of the Western blots are shown in Figure S11.

In A2780, the expression of *survivin* was reduced by the combination of panobinostat and cisplatin and by luminespib alone. Panobinostat alone and the combination of panobinostat and luminespib with or without cisplatin increased the expression of *survivin*. Only treatment with luminespib alone did not increase the expression of *APAF-1*; all other treatments resulted in a small increase in expression. The expression of the tumor suppressor gene *p21* was generally increased by each treatment. Treatment with panobinostat or luminespib alone (including the combination of both) had only a weak effect on the expression of *p21*. The expression of *p21* was strongly induced by any treatment regimen containing cisplatin (Figure 5A). In A2780CisR, the expression of *survivin* was reduced by cisplatin alone and cisplatin in combination with panobinostat or luminespib. Combination treatment with panobinostat and luminespib (also with cisplatin) also led to a significant reduction in *survivin* expression. A significant increase in the expression of *APAF-1* could only be achieved by treatment with cisplatin alone. *p21* Expression was increased in A2780CisR for all treatment regimens containing either cisplatin or panobinostat. Luminespib alone had no effect on the expression of *p21* (Figure 5B). To confirm the results of mRNA gene expression, protein expression of *p21, APAF-1,* and *survivin* was analyzed by Western blot (Figure 6). 

*APAF-1*, *p21,* and *survivin* expressions were strongly influenced by our treatments. Dual or triple combinations containing panobinostat resulted in both cell lines in downregulation of survivin and upregulation of *p21* and *APAF-1* in comparison to a single treatment with cisplatin. In contrast, dual combinations with luminespib were clearly less effective and showed the desired effects only in combination with panobinostat.

### 2.5. Effects of Panobinostat or HSP990 Plus Cisplatin on Cell Viability in High Grade Serous Ovarian Cancer Cell Lines CaOV3 and OVCAR3 and Their Cisplatin-Resistant Sub-Cell Lines

To demonstrate that our findings are not limited to the ovarian cancer cell pair A2780/A2780CisR, the experimental conditions yielding the largest shift factors in MTT assays consisting of dual combinations panobinostat plus cisplatin and HSP990 plus cisplatin (Table 2) were applied to the high grade serous ovarian cancer (HGSOC) cell lines CaOV3 and OVCAR3, as well as their cisplatin resistant sublines. Table S3 shows IC_50_ values from single treatments with panobinostat and HSP990. IC_50_ values of panobinostat and HSP990 in CaOV3, OVCAR3 and their cisplatin-resistant sub-lines (Table 3) are in a similar range as IC_50_ values at A2780 and A2780CisR cell lines (Table 1). 

The applied dual combinations increased the cisplatin potency significantly in all cell lines with shift factors up to 4.7 except HSP990 plus cisplatin in OVCAR3 where no significant shift was observed (Table 4 and Table S4). Notably, 10 nM panobinostat was able to completely reverse the cisplatin resistance of CaOV3CisR (CaOV3, IC_50 (cisplatin)_: 1.92 µM; CaOV3CisR, IC_50 (cisplatin + 48h panobinostat preincubation)_: 1.38 µM; Table 4 and Table S4). 

### 2.6. Effects of HDACi or HSP90i and Cisplatin on the Non-Cancer Cell Line HEK293

**Figure 7:**
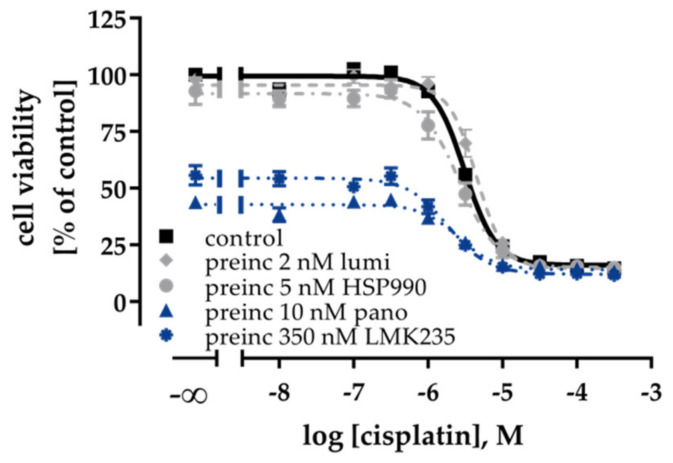
48 h preincubation with luminespib, HSP990, panobinostat or LMK235 prior to 72 h cisplatin treatment did not affect cisplatin sensitivity of the non-cancerous cell line HEK293. Data shown are mean ± SEM of three independent experiments. Results (IC_50_, pIC_50_, SEM, and shift factors) are summarized in Table S6.

Next, to investigate if the observed effects were tumor specific in nature, we treated the non-cancerous cell line HEK293 with a combination of HDACi or HSP90i with cisplatin (Figure 7). Both HSP90i did not affect the cell viability, whereas the HDACi reduced the cell viability around 50%, which can be recognized by the lowering of the top plateau of the concentration effect curves. Notably, none of the inhibitors significantly increased the potency of cisplatin in HEK293 cells, except LMK235 which slightly (1.7 fold) reduced the IC_50_ of cisplatin (Figure 7 and Table S6). Therefore, we assume that the observed effects of HSP90i and HDACi on cisplatin have a certain selectivity for ovarian cancer cells over non-tumor cells.

### 2.7. Long Term Treatment with Low-Dose HDACi or HSP90i can Overcome and Prevent the Development of Cisplatin Resistance in A2780 Ovarian Cancer Cells.

**Figure 8:**
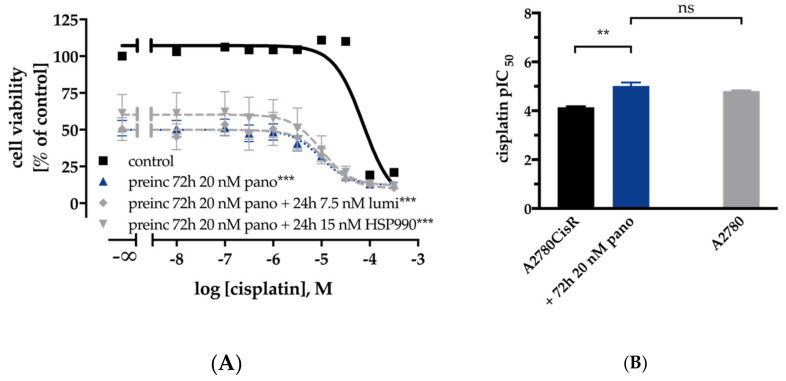
Effects of prolonged (72 h instead of 48 h) preincubation with HDACi / HSP90i on cisplatin sensitivity in A2780CisR ovarian cancer cells. 72 h preincubation with panobinostat or combination of 72 h of panobinostat with 24 h of luminespib or HSP990 prior to 48 h cisplatin treatment increased cisplatin sensitivity in A2780CisR (**A**). pIC_50_ ± SEM values of cisplatin in A2780CisR using 72 h preincubation with panobinostat compared to A2780 (**B**). Results (IC_50_, pIC_50_, SEM, and shift factor) are summarized in Table S5. Statistical analysis was performed using t-test. Levels of significance: ns *p* > 0.05, ** *p* ≤ 0.01, *** *p* ≤ 0.001.

The development of cisplatin resistance is a major obstacle in the therapy of ovarian cancer. We observed favorable effects with dual combinations (HSP90i or HDACi and cisplatin) on the cisplatin sensitivity in A2780 cells. In A2780CisR cells, the chemosensitizing effect on cisplatin was less pronounced. Therefore, we were interested in whether a prolongation of the incubation time of HDACi or HSP90i had an influence on cisplatin sensitivity in A2780CisR. Instead of 48 h, we applied a 72 h preincubation with HDACi followed by 48-h cisplatin incubation or 48 h preincubation with HDACi followed by 24 h preincubation with HDACi plus HSP90i and subsequent addition of cisplatin for 48 h. The results of MTT assays for A2780CisR with this incubation scheme are shown in Figure 8.

Extension of the preincubation time of HDACi from 48 (as applied in Figures S6 and S7) to 72 h resulted in only slightly decreased shift factors in A2780 cells (Figure S9, Table S5). In contrast, prolongation of the incubation time increased shift factors in A2780CisR cells and reduced the cell viability (Figure 8 and Figure S9A,B and Table S5B). As a result of the prolonged incubation scheme, cisplatin IC_50_ control values differed from those reported in Figures S6 and S7. 

**Figure 9:**
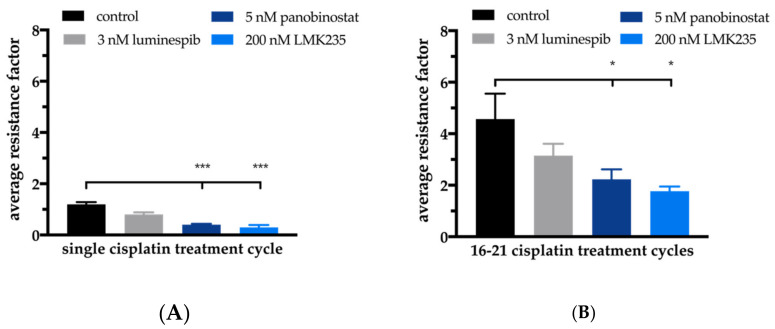
Effects of HDACi or HSP90i on short-term (**A**) or long-term (**B**) cisplatin stress-induced changes of cisplatin sensitivity in A2780 ovarian cancer cells. Average resistance factors of cisplatin after single cisplatin treatment cycle (“cisplatin stress”) (A) or after 16–21 cisplatin treatment cycles (averaged data over Treatment Cycles 16–21) in A2780 cells. Cisplatin treatment cycle means treatment for 6 h with an IC_50_ of cisplatin alone (“control”) or in permanent presence of 3 nM luminespib, 5 nM panobinostat or 200 nM LMK235. Resistance factors were calculated by dividing the IC_50_ of cisplatin obtained after a treatment cycle (“stressed” cells) by the IC_50_ of cisplatin from unstressed A2780 cells. Statistical analysis was performed using t-test. Levels of significance: * *p* ≤ 0.05, *** *p* ≤ 0.001.

The prolonged incubation scheme resulted in an IC_50_ for cisplatin of 72.7 µM for A2780CisR. Preincubation for 72 h of panobinostat enhanced the cisplatin potency 7.6-fold, resulting in a cisplatin IC_50_ of 9.56 µM (Figure 8B). This indicates complete reversal of cisplatin resistance, as can be seen from the cisplatin IC_50_ of 15.4 µM for A2780 cells using the prolonged MTT pretreatment scheme (Figure 8B). The complete reversal of cisplatin resistance by panobinostat preincubation in A2780CisR and the observed hypersensitization in A2780 cells prompted us to investigate the effect of a permanent presence of the HDACi panobinostat or LMKM235 or the HSP90i luminespib on cisplatin sensitivity. This was studied in short-term cisplatin-treated A2780 cells (Figure 9A, single cisplatin treatment cycle) as well as in long-term cisplatin-treated cells (Figure 9B, 16–21 cisplatin treatment cycles).

Treatment cycle means that A2780 cells were exposed to cisplatin in an IC_50_ for 6 h followed by washout, a two-day recovery and subsequent determination of cisplatin IC_50_ by MTT. Single cisplatin treatment cycle applied to A2780 cells resulted in approximately the same IC_50_ value of cisplatin as obtained without cisplatin stress. Notably, the same cisplatin treatment cycle applied to A2780 cells in permanent presence of 3 nM luminespib, 5 nM panobinostat or 200 nM LMK235 resulted in reduced IC_50_ values indicating (hyper)sensitization towards cisplatin (Figure 9, “resistance” factors of less than 1). Increased sensitivity towards cisplatin was significant for both HDACi (5 nM panobinostat and 200 nM LMK235; Figure 9), confirming the data shown in Figure 2 and Figure S5 where we were able to sensitize A2780 cells towards cisplatin by HDACi pretreatment. Long-term intermittent treatment with IC_50_ of cisplatin (16-21 treatment cycles) resulted in the resistant subclone A2780CisR with a resistance factor of around 4.5. When this long-term intermittent treatment with cisplatin was performed in the presence of HDACi or HSP90i, resistance development was moderately (but not significantly) reduced by 3 nM luminespib. Notably, in permanent presence of 5 nM panobinostat or 200 nM LMK235, a significant reduction of the resistance development was achieved. After 21 weekly treatment cycles, the resistance factor of cisplatin was only 1.7 in presence of LMK235, demonstrating that the development of cisplatin resistance was almost completely abrogated in the presence of HDACi. 

## 3. Discussion

This study reports on single and combined effects of HDACi and HSP90i with cisplatin in human ovarian cancer cell lines and offers a strategy to address chemoresistance in ovarian cancer that is urgently needed [13]. In 2007, Solár et al. reported that geldanamycin, one of the first discovered HSP90i, increased the sensitivity to cisplatin of the cisplatin-resistant ovarian cancer cell line A2780CisR [40]. Similarly, HSP90i have recently been shown to reverse cisplatin resistance in human ovarian cancer cells [21]. However, these findings have not yet led to clinical approval of drugs for reversal of chemoresistance. HDACi are approved epigenetic drugs for the treatment of specific lymphomas, e.g., cutaneous T cell lymphoma or multiple myeloma. Their potential to reverse chemoresistance of DNA-damaging drugs is currently explored by many groups including ours [31,32,33,41,42]. Weberpals et al. could show that the increase in cisplatin sensitivity by HDACi could be caused by a decrease in the expression of BRCA1 [43]. This reduced expression is achieved by HDACi alone or more strongly in combination with cisplatin. This suggests a therapeutic option for patients with tumors expressing significant levels of BRCA1. Additionally, there is the possibility to combine different epigenetic therapy strategies. For example, another group has been successful in overcoming cisplatin resistance of an ovarian cancer cell line by combining DNA methyltransferase inhibitors (DNMTi) and HDACi [44]. Furthermore, it has been shown that this combination leads to cell cycle arrest and increased apoptosis rate [45]. For the DNMTi decitabine, a BRCA1 related mechanism of action is discussed. These and our results in the field of epigenetic therapy of ovarian cancer in cellular models underline the importance of research in this field. To the best of our knowledge, the combination of HSP90i with HDACi and cisplatin has not yet been thoroughly studied in ovarian cancer and was thus the aim of this project. 

We could confirm the results from previous studies [21,40] that pretreatment with HSP90i led to an increase in cisplatin potency (Figure S6A,B and Table 2, Table S3A and Table 4). However, in contrast to Zhang et al., the sensitizing effect of HSP90i was only seen in A2780 but not in the cisplatin-resistant cell line A2780CisR although HSP90 inhibition led to a decrease in AKT expression and phosphorylation in both cell lines (Figure 1C) which is in accordance with literature reports [46]. In contrast to A2780/A2780CisR cells, we observed a larger cisplatin-sensitizing effect after HSP990 preincubation in cisplatin-resistant OVCAR3CisR than in OVCAR3 cells (Table 2, Table S3A and Table 4). Further, we could demonstrate that the sequence of inhibitor incubation matters: only pretreatment with HSP90i prior to cisplatin addition but not coincubation with cisplatin increased cisplatin potency (Figure S6A,B and Table 2 and Table S3). 

Protein levels of HDAC isoforms in A2780 cells were not different in our previously shown studies [33] compared to those of Khabele et al. [30]. Consequently, combinations of the pan-HDACi panobinostat or LMK235 with cisplatin had similar synergistic effects as recently reported [32,33,42] (Figure S6C,D and Table 2 and Table S3A). Panobinostat and LMK235 showed no major differences, and, in addition to synergistic effects with cisplatin (Figure S6), both HDACi reduced cell proliferation significantly in A2780CisR but not in A2780 (Figure 1A,B), although an increase in acetylation was observed in both cell lines (Figure 1D). This is however in accordance with reports that resistant cancer cells undergo further epigenetic changes and might thus be more susceptible to HDACi [47]. Combinations of HDACi and cisplatin were synergistic as shown by the gold standard method of Chou-Talalay [32,33,48]. Of note, both coincubation of HDACi with cisplatin and 48 h preincubation with HDACi prior to addition of cisplatin increased cisplatin potency. However, preincubation was more effective in resistant A2780CisR, CaOV3CisR and OVCAR3CisR (Table 2 and Table 4), which is somewhat different from the results of Ong et al. who showed marked increases in sensitivity to cisplatin by coincubation of vorinostat with cisplatin [49].

The importance of the sequence of inhibitor addition in combination experiments was further explored when combining HDACi and HSP90i. Preincubation with HDACi prior to addition of HSP90i increased HSP90i potency in A2780 and A2780CisR (Figure S7) up to three-fold, whereas, vice versa, preincubation with HSP90i prior to addition of HDACi did not or only slightly increase the potency of HDACi (Figure 2A–D and Figure S4 and Table S1). These results are in accordance with data from Kaiser et al. (myeloma cells) and Kim et al. (anaplastic thyroid carcinoma cells) showing that luminespib was more potent and synergistic after preincubation with HDACi [29,34]. Synergy improves the efficiency of a therapy and could also help to reduce employed concentrations, thus avoiding side effects limiting clinical application [50]. Maximum concentrations used in our study (10 nM luminespib, 15 nM HSP990, and 25 nM panobinostat) were far below the maximum plasma levels resulting from application of the maximum tolerated dose (2.4 µM for luminespib, 1.3 µM for HSP990 and 721 nM for panobinostat) [51,52,53]. Preincubation with HDACi leads to hyperacetylation of histone and non-histone proteins, including HSP90 [54], mediated at least by HDAC6 and HDAC10 [35]. Increase in HSP90 acetylation then results in HSP90 inhibition, which is further boosted by addition of HSP90i [54], thus explaining the synergistic behavior of combinations of HDACi and HSP90i (Figure 2 and Figure S5). Since the contribution of different HDAC subtypes to the effect of HSP90i is not completely understood and an HDAC6-selective inhibitor showed no superior effect over pan-HDACi in increasing the chemosensitivity of cisplatin [33,41], we decided to use the pan-HDACi panobinostat and LMK235 [32]. 

Based on the promising results of dual combinations of HDACi and HSP90i in ovarian cancer, we evaluated the potential of triple combinations of HDACi, HSP90i and cisplatin. As can be seen in Figure S7 and Table 2 and Table S3B,C, triple combinations were not superior to dual combinations of HSP90i and cisplatin or HDACi and cisplatin, regardless of the order of application of HDACi, HSP90i and cisplatin. Between almost all applied combinations (dual and triple combinations) were no significant differences in IC_50_ values of cisplatin in A2780 or A2780CisR (Table 2). However, the best effects of HDACi in increasing cisplatin potency (highest shift factors) were observed in A2780 and not in A2780CisR (Table S3B,C). In A2780 cells, the largest increase in cisplatin potency was obtained by 48 h preincubation with HSP990 (shift factor: 6.7), panobinostat (shift factor: 5.9) or LMK235 (shift factor: 5.9). In A2780CisR cells, neither combination led to a complete reversal of cisplatin resistance, except if the preincubation time of HDACi was extended from 48 to 72 h (Figure 8 and Table S5). In contrast, 48 h preincubation with panobinostat was sufficient for complete reversal of cisplatin resistance in the HGSOC cell line CaOV3CisR (Table 3). HSP90i increased cisplatin sensitivity but did not overcome (completely reverse) cisplatin resistance in A2780CisR, CaOV3CisR or OVCAR3CisR. High HSP90 expression is associated with drug resistance [55], however, A2780CisR cells did not show increased HSP90 expression compared to A2780 (Figure 1C,D). In addition, in a previous study, we were also not able to reverse a fully established cisplatin resistance by various kinase inhibitors [26]. Since an extended preincubation time of panobinostat (72 h instead of 48 h) led to a complete reversal of cisplatin resistance (Figure 8), we were interested in studying the effects of long-term, sub-cytotoxic concentrations of panobinostat on the development of chemoresistance against cisplatin. For comparison, the HSP90i luminespib was included. Chemoresistance against cisplatin was induced as previously described [26,38,56]. Cells were exposed to IC_50_ of cisplatin for 6h followed by washout and recovery for one week (termed as “treatment cycle”). We demonstrated that one treatment cycle in continuous presence of low concentrations of HDACi (5 nM panobinostat or 200 nM LMK235) significantly increased the potency of cisplatin as noticed by a resistance factor of less than 1 (Figure 9A). The HSP90i luminespib also led to an increase in cisplatin potency but this effect was not significant. Of note, long-term incubation of low concentrations of HDACi for up to 21 treatment cycles prevented almost completely the development of cisplatin resistance (Figure 9B). Permanent presence of 200 nM LMK235 gave a resistance factor of 1.7, whereas, in the absence of LMK235, a resistance factor of 4.5 was obtained. This shows that epigenetic modulation by HDACi is able to prevent the development of chemoresistance against cisplatin. Interestingly, these data are in accordance with results previously published by us using resveratrol or ellagic acid (which have also been described as HDACi) to prevent the development of resistance against cisplatin [26].

HDACi are known to induce apoptosis in ovarian cancer cells by modulating the expression of genes regulating cell growth, cell cycle progression or apoptosis [57]. Apoptosis induction by HSP90i has also been described for various cancers [58]. We were thus interested in the regulation of key genes of apoptosis, cell survival, and cell cycle by HSP90i, HDACi, and cisplatin. Proapoptotic *APAF-1* is upregulated by HDACi in hepatocellular carcinomas [59]. Overexpression of *survivin* is known to be associated with poor prognosis and high-grade cancers [60]. A high expression of *p21* could be a predictor of cisplatin sensitivity in ovarian cancer [61]. We thus examined expression of the key genes *APAF-1, survivin,* and *p21* in PCR and Western blot (Figure 3, Figure 4, Figure 5 and Figure 6). 

Effects were most pronounced in A2780CisR in Western blot. Survivin expression was even increased upon treatment with cisplatin or luminespib (or their combination) in A2780CisR (Figure 5), possibly explaining the lower effect of HSP90i in cisplatin resistant cells (Table 2). However, panobinostat and any combination thereof, reduced expression of survivin, giving a further hint to the stronger effect of HDACi over HSP90i. p21 expression was increased upon panobinostat (or panobinostat-containing combinations) but unaffected by luminespib and only slightly increased under cisplatin treatment, again explaining superior effects of HDACi in inducing cell cycle arrest via p21 followed by apoptosis or caspase 3/7 activation (Figure 4, Figures S6E, S7C and S8). *APAF-1* regulation was affected by the modulators similar as p21. Luminespib gave almost no increase in *APAF-1*, whereas panobinostat and cisplatin increased APAF-1 in A2780CisR cells (Figure 3, Figure 5 and Figure 6). Taken together, the effects of HDACi on gene expression of survivin, p21, and APAF-1 may explain increased apoptosis and caspase activation including synergy with the DNA-damaging agent cisplatin. Further, effects on gene expression by panobinostat observed in this study are in accordance with previous results from our group [33]. Interestingly, the triple combination of HDACi, HSP90i and cisplatin is less advantageous than the dual combination HDACi and cisplatin regarding increase in *APAF-1* and *p21* and decrease in survivin. The dual combination HSP90i and cisplatin is not favorable at all regarding gene expressions of *APAF-1, p21* and *survivin*. 

In conclusion, this study revealed that preincubation with HDACi increase the potency of HSP90i in sensitive and cisplatin-resistant ovarian cancer cells but not vice versa. Further, preincubation with HDACi or HSP90i prior to cisplatin increase cisplatin sensitivity and act synergistically. HDACi are more effective than HSP90i in cisplatin-resistant cell lines. Surprisingly, triple combinations of HSP90i, HDACi and cisplatin were not superior to dual combinations of HDACi and cisplatin. Finally, permanent presence of low concentrations of HDACi significantly impedes the development of cisplatin resistance. Thus, combinations of HDACi and HSP90i as well as combinations of HDACi and cisplatin may improve the therapeutic outcome of ovarian cancer, in particular of HGSOC.

## 4. Materials and Methods

### 4.1. Materials 

Materials and reagents for cell culture were purchased from PAN Biotech (Aidenbach, Germany) unless otherwise stated. Cisplatin was from Sigma-Aldrich (Steinheim, Germany) and 3-(4,5-dimethylthiazol-2-yl)-2,5-diphenyltetrazolium bromide (MTT) from Serva (Heidelberg, Germany). Luminespib and HSP990 were gifts from Novartis, Basel, Switzerland. Panobinostat was purchased from Selleckchem (Eching, Germany). LMK235 was synthesized in the group of Prof. T. Kurz, Heinrich Heine University, Duesseldorf, Germany.

### 4.2. Cell Lines

The human embryonic kidney cells HEK293 was obtained from the German Collection of Microorganism and Cell Cultures (DSMZ, Braunschweig, Germany). The human ovarian carcinoma cell lines CaOV3 and OVCAR3 were obtained from American Type Culture Collection (ATCC, Manassas, Virginia, USA). The human ovarian carcinoma cell line A2780 was obtained from European Collection of Cell Cultures (ECACC, Salisbury, UK). The cisplatin resistant sublines A2780CisR, CaOV3CisR, and OVCAR3CisR were generated by exposing the parental cell line to weekly cycles of cisplatin in an IC_50_ concentration according to Gosepath et al. [56]. Results of STR analysis of A2780 and A2780CisR can be found in Table S7. Both cell lines have been previously characterized in our group [25,26]. Cisplatin resistant cell lines in the presence of an inhibitor were generated in an analogous manner in the permanent presence of 3 nM luminespib, 5 nM panobinostat or 200 nM LMK235. 

Cells were grown at 37 °C under humidified air supplemented with 5% CO_2_ in RPMI 1640 (A2780, CaOV3 and, OVCAR3) or DMEM (HEK293) containing 10% heat inactivated fetal calf serum, 120 IU/mL penicillin, and 120 µg/mL streptomycin.

### 4.3. Cell Viability Assay

The cytotoxic activity of the inhibitors and cisplatin was evaluated by an MTT assay as previously described [26,32,33]. To analyze the interactions between HSP90i and HDACi, cells were preincubated with one of these inhibitors for 48h followed by treatment for 72 h together with the second inhibitor. To determine the effect of HSP90i and HDACi on cisplatin sensitivity, inhibitors were coincubated for 72 h with cisplatin or preincubated for 48 h prior to cisplatin addition. Total incubation time of cells never exceeded 120 h. All experiments were performed at least three times in triplicates. Shift factors (SF) were calculated by dividing the IC_50_ value from single agent-treated cells by the IC_50_ value of combined treated cells. Resistance factors (RF) were calculated as the ratio between IC_50_ values of cisplatin-resistant cells and the sensitive subline.

### 4.4. Doubling Time

A2780 and A2780CisR cells were plated in 24-well plates (Sarstedt AG, Nürmbrecht, Germany) and incubated under culture conditions for 24 h. After 24 h, initial cell number was determined and cells were incubated with HSP90i or HDACi for additional 24, 48, and 72 h. After each time point, cells were harvested by trypsin-EDTA, diluted in 0.9% NaCl with 0.1% NaN_3_ (Acros Organics, Geel, Belgium) and counted by flow cytometry (Partec GmbH, Muenster, Germany). Doubling time was calculated using GraphPad Prism v. 4.0 (GraphPad Software Inc, San Diego, USA).

### 4.5. Analysis of Apoptosis Induction

To determine the number of apoptotic cells, A2780 and A2780CisR cells were treated in 24-well plates with the indicated concentrations of the inhibitors for 24 or 48 h. If mentioned, cells were first treated for 48 h with indicated inhibitor and then treated for 6 h with the IC_50_ of cisplatin. Next, cells recovered for 24 h. After total incubation time, cells were lysed overnight in hypotonic staining buffer (0.1% Triton X-100, 0.1% sodium citrate in sterile water) containing 100 µg/mL propidium iodide (PI, PromoCell, Heidelberg, Germany) and analyzed by flow cytometry (Partec GmbH, Muenster, Germany). Ten percent DMSO incubated for 24 h served as positive control for apoptosis induction.

### 4.6. Immunoblotting

Cells were treated with the inhibitors for the indicated time periods. After treatment, cells were lysed by using Lysis Buffer 6 (R&D Systems Inc, Minneapolis, MN, USA). Protein concentration was determined with the bicinchoninic acid (BCA) protein assay. From 20 to 30 µg of protein were separated by SDS-PAGE (8% polyacrylamide gel for AKT and pAKT, 12% polyacrylamide gel for α-Tubulin and Acetyl-α-Tubulin) and transferred to PVDF membrane (Millipore Corporation, Billerica, MA, USA). Blots were incubated with primary antibodies against HSP90 α/β (Cat. No. sc-7947), AKT (Cat. No. sc-8312), phospho AKT (5.Ser 473, Cat. No. sc-293125), β-actin (Cat. No. sc-47778), α-tubulin (Cat. No. sc-8035) (Santa Cruz Biotechnology, Heidelberg, Germany), APAF-1 (Cat. No. MAB868), survivin (Cat. No. AF886) and p21 (AF1047) (Bio-Techne GmbH, Wiesbaden-Nordenstadt, Germany). Membranes were incubated with the corresponding HRP-conjugated secondary antibody (R&D Systems Inc, Minneapolis, MN, USA). Proteins were visualized using luminol reagent (Santa Cruz Biotechnology, Heidelberg, Germany) and an INTAS Science Imaging Instrument (Chemo Imager INTAS, Goettingen, Germany).

### 4.7. RNA Extraction and PCR

Cells were treated with the indicated conditions and harvested at 70–80% confluence. Total RNA extraction was performed using RNeasy Mini Kit with DNase treatment according to the manufacturer’s instructions (Qiagen, Hilden, Germany). Total RNA (µg) was reverse transcribed using the High Capacity cDNA Reverse Transcription Kit (Thermo Scientific, Wesel, Germany). Thermocycler program setting was initiated for 10 min at 25 °C, followed by 120 min at 37 °C. In total, 20 µg cDNA were diluted in 100 µL TE buffer (1 mM Tris-HCl, 0.1 mM EDTA in distilled water; pH 7.50). Specific primers (Sigma Aldrich, Steinheim, Germany, Table 5) were designed by Primer-BLAST (NIH, Bethesda, Maryland, USA) [62]. The PCR program consisted of an initial denaturation step at 95 °C for 2 min for the first cycle then continued with 94 °C for 20 s, 57 °C for 30 s and 72 °C for 60 s for 40 cycles. The PCR products were separated using 2% agarose gel in TAE buffer (2.42 g Tris, 0.57 mL acetic acid and 185 mg EDTA disodium salt in 500 mL distilled water). DNA bands were stained using GelRed (New England Biolabs, Frankfurt, Germany) and detected with an Intas Gel iX Imager UV system (INTAS, Goettingen, Germany). GeneRuler 50 bp (Thermo Scientific, Wesel, Germany) was used as DNA ladder and *β-tubulin* served as housekeeping gene. 

### 4.8. Caspase 3/7 Activation Assay

Compound-induced activation of caspases 3 and 7 was analyzed using the CellEvent Caspase-3/7 green detection reagent (Thermo Scientific, Wesel, Germany) according to the manufacturer´s instructions. Briefly, A2780CisR cells were seeded in 96-well plates (Corning, Kaiserslautern, Germany) at a density of 4000 *c/w*. Cells were treated with panobinostat or LMK235 and/or HSP990 or luminespib (as indicated) for a maximum treatment duration of 48 h. Then, medium was removed and 50 µL of CellEvent Caspase 3/7 green detection reagent (2 µM in PBS supplemented with 5% heat inactivated FBS) was added. Cells were incubated for 30 min at 37 °C in a humidified incubator before imaging by using the Thermo Fisher ArrayScan XTI high content screening (HCS) system with a 10 X magnification (Thermo Scientific). Hoechst 33342 was used for nuclei staining.

### 4.9. Statistical Methods

Assays were performed at least in three independent experiments. Concentration–effect curves were obtained using Prism 4.0 from GraphPad, San Diego, CA, USA, by fitting the pooled data to the four-parameter logistic equation with variable hill slope. Flow cytometry data were analyzed using FloMax 2.82 (Partec, Muenster, Germany). Statistical significance was assessed by two-tailed Student´s t-test. 

## Figures and Tables

**Table 1:** Cytotoxic activity of panobinostat, LMK235, luminespib and HSP990.

Cell Line	HDACi				HSP90i			
	Panobinostat		LMK235		Luminespib		HSP990	
	IC_50_ [nM]	pIC_50_ ± SEM	IC_50_ [nM]	pIC_50_ ± SEM	IC_50_ [nM]	pIC_50_ ± SEM	IC_50_ [nM]	pIC_50_ ± SEM
**A2780**	28.0	7.55 ± 0.02	847	6.07 ± 0.03	11.2	7.95 ± 0.06	20.1	7.70 ± 0.06
**A2780CisR**	28.2	7.55 ± 0.03	644	6.19 ± 0.02	16.4	7.78 ± 0.04	29.8	7.53 ± 0.02

Cell viability was determined by MTT assay after a 72 h incubation. Data shown are the mean of pooled data from at least three experiments each carried out in triplicate. Concentration effect curves are shown in Figure S2A,B.

**Table 2:** Influence of dual or triple combinations with HDACi or HSP90i on the cytotoxic activity of cisplatin in A2780 and A2780CisR cells.

**A – Dual Combination (inhibitor + cDDP)**									
**cell line**	**cDDP IC50 [µM]**								
	**Control** **(cDDP only)**	**HSP90i**				**HDACi**			
		**Luminespib**		**HSP990**		**Panobinostat**		**LMK235**	
		**Coinc**	**preinc**	**coinc**	**preinc**	**coinc**	**preinc**	**coinc**	**preinc**
**A2780**	3.34	2.41	0.71	2.22	0.50	1.63	0.57	0.69	0.57
**A2780CisR**	19.7	24.9	13.2	25.7	11.8	16.6	6.53	11.0	7.05
**B – Triple Combination I (HSP90i prior to HSP90i + HDACi + cDDP)**									
		**A2780** **cDDP IC_50_ [µM]**				**A2780CisR** **cDDP IC_50_ [µM]**			
		**cDDP**	**HDACi/cDDP**			**cDDP**		**HDACi/cDDP**	
			**pano**	**LMK235**				**pano**	**LMK235**
**HSP90i**	**lumi**	0.71	0.65	0.63		13.2		12.3	9.69
	**HSP990**	0.50	0.70	0.95		11.8		7.78	7.07
**C – Triple Combination II (HDACi prior to HDACi + HSP90i + cDDP)**									
		**A2780** **cDDP IC_50_ [µM]**				**A2780CisR** **cDDP IC_50_ [µM]**			
		**cDDP**	**HSP90i/cDDP**			**cDDP**		**HSP90i/cDDP**	
			**lumi**	**HSP990**				**lumi**	**HSP990**
**HDACi**	**pano**	0.57	0.91	0.84		6.53		6.07	5.91
	**LMK235**	0.57	0.70	0.84		7.05		9.23	9.26

Data shown are IC_50_ values in µM obtained from three independent experiments each carried out in triplicate: (**A**) dual combinations in A2780 and A2780CisR; and (**B**,**C**) triple combinations in A2780 and A2780CisR. Preincubation means a 48 h preincubation with the indicated inhibitor followed by a 72-h incubation with cisplatin (**A**) or cisplatin plus indicated HDACi/HSP90i (**B**,**C**). pIC_50_ and SEM are shown in Table S2. Control denotes the IC_50_ value for cisplatin without inhibitor treatment. Concentrations used are 5 nM luminespib, 10 nM HSP990, 500 nM LMK235, and 10 nM panobinostat in A2780 and 10 nM luminespib, 15 nM HSP990, 700 nM LMK235, and 20 nM panobinostat in A2780CisR. (**A**) IC_50_ values are derived from the results in Figure S6A–D.

**Table 3:** Cytotoxic activity of panobinostat and HSP990 in sensitive and cisplatin-resistant HGSOC cell lines CaOV3 and OVCAR3.

Cell Line	Panobinostat		HSP990	
	IC_50_ [nM]	pIC_50_ ± SEM	IC_50_ [nM]	pIC_50_ ± SEM
**CaOV3**	15.9	7.80 ± 0.02	40.9	7.39 ± 0.03
**CaOV3CisR**	16.9	7.77 ± 0.02	55.6	7.26 ± 0.02
**OVCAR3**	41.3	7.38 ± 0.02	27.6	7.56 ± 0.03
**OVCAR3CisR**	30.5	7.52 ± 0.01	25.0	7.60 ± 0.02

Cell viability was determined by MTT assay after a 72 h incubation. Data shown are the mean of pooled data from at least three experiments each carried out in triplicate. Concentration effect curves are shown in Figure S2C–F.

**Table 4:** Influence of dual combinations with panobinostat or HSP990 on the cytotoxic activity of cisplatin in CaOV3, OVCAR3, and their cisplatin resistant sublines.

Cell Line	Control (Cisplatin Only)	+ 48h Pretreatment			
		HSP990		Panobinostat	
		Cisplatin IC_50_ [µM]	SF	Cisplatin IC_50_ [µM]	SF
**CaOV3**	1.92	0.79	2.4 ***	1.04	1.8 ***
**CaOV3CisR**	4.80	3.20	1.5 **	1.38	3.5 ***
**OVCAR3**	3.94	2.77	1.4 ns	1.50	2.6 **
**OVCAR3CisR**	37.7	12.6	3.0 *	8.03	4.7 ***

Data shown are IC_50_ values and shift factors (SF) obtained from three independent experiments each carried out in triplicate. The concentrations used were 10 nM for panobinostat and 10 nM HSP990. SF were calculated as the ratio of IC_50_ of cisplatin and the IC_50_ of the corresponding drug combination. pIC_50_ and SEM are shown in Table S4. Statistical analysis was performed using t-test. Levels of significance: ns *p* > 0.05, * *p* ≤ 0.05, ** *p* ≤ 0.01, *** *p* ≤ 0.001.

**Table 5:** Primer sequences for PCR.

Gene.	Primer Forward	Primer Reverse	Size [bp]
*BAK*	GAACAGGAGGCTGAAGGGGT	TCAGGCCATGCTGGTAGACG	307
*survivin*	CGAGGCTGGCTTCATCCACT	ACGGCGCACTTTCTTCGCA	283
*Bcl-xL*	CTGAATCGGAGATGGAGACC	TGGGATGTCAGGTCACTGAA	211
*BAX*	GATGCGTCCACCAAGAAGCT	CGGCCCCAGTTGAAGTTG	170
*β-tubulin (TUBB)*	TCTACCTCCCTCACTCAGCT	CCAGAGTCAGGGGTGTTCAT	161
*Mcl-1*	GGACATCAAAAACGAAGACG	GCAGCTTTCTTGGTTTATGG	154
*APAF-1*	ACAATGCTCTACTACATGAAGGATATAAAGA	CACTGGAAGAAGAGACAACAGGAA	81
*p21*	CCTAATCCGCCCACAGGAA	AAGATGTAGAGCGGGCCTTTG	68

## Supplementary Materials

The following are available online at https://www.mdpi.com/1422-0067/21/21/8300/s1, Figure S1: Effects of HSP90i and HDACi on cell growth and protein expression, Figure S2: Antiproliferative activity of HDACi and HSP90i against ovarian cancer cell lines, Figure S3: Concentration-dependent 48 h incubation with HSP990 (IC50, three-fold and five-fold IC50) and time-dependent incubation with three-fold IC50 of HSP990, Figure S4: Influence on the cytotoxicity of HSP90i and HDACi on each other, Figure S5: Apoptosis induction was analyzed in A2780 and A2780CisR after a 24 h incubation with an HSP90i or panobinostat followed by a 24 h incubation with both inhibitors in combination, Figure S6: HSP90i and HDACi treatment enhance the activity of cisplatin in A2780 and A2780CisR, Figure S7: Triple combination treatment (HDACi, HSP90i, cisplatin) is not superior to dual combination of HDACi and cisplatin with regard to cisplatin cytotoxicity and apoptosis induction, Figure S8: Effect of HDACi, HSP90i and drug combinations on cell cycle distribution, Figure S9: A 72 h preincubation with LMK235 (350 nM for A2780 and 500 nM for A2780CisR) (A,B) or panobinostat (10 nM for A2780 and 20 nM for A2780CisR) (C) or 48 h preincubation with HDACi followed by 24 h incubation together with HDACi and HSP90i increased cisplatin sensitivity after 48 h cisplatin treatment at A2780 (C) and A2780CisR (A,B) cells, Figure S10: Effects of HDACi or HSP90i incubation or preincubation prior to cisplatin on apoptosis related genes, Figure S11: Effects of HDACi or HSP90i incubation or preincubation prior to cisplatin on protein expression levels, Table S1: Influence on the cytotoxicity of HSP90i and HDACi on each other, Table S2: Influence of dual or triple combinations with HDACi or HSP90i on the cytotoxic activity of cisplatin in A2780 and A2780CisR cells, Table S3: Shift factors of dual or triple combinations with HDACi or HSP90i on the cytotoxic activity of cisplatin in A2780 and A2780CisR cells, Table S4: Influence of dual combinations with panobinostat or HSP990 on the cytotoxic activity of cisplatin in CaOV3, CaOV3CisR, OVCAR3, and OVCAR3CisR cells, Table S5: Effects of short-term treatment with HDACi or HSP90i on cisplatin sensitivity of A2780/A2780CisR ovarian cancer cells, Table S6: Influence of HDACi/HSP90i preincubation on cisplatin sensitivity of non-tumor cell line HEK293, Table S7: Results of STR analysis of A2780 and A2780CisR.

## Abbreviations

(p)Akt/AKT	(phosphorylated) protein kinase B
APAF-1	apoptotic protease activating factor 1
BAK	Bcl-2 homologous antagonist killer
BAX	Bcl-2-associated X protein
Bcl-xL	B-cell lymphoma-extra large
cDDP	*cis*-diamminedichloroplatinum(II) (cisplatin)
coinc	coincubation
HDAC	histone deacetylase
HDACi	histone deacetylase inhibitor
HSP90	heat shock protein 90
HSP90i	heat shock protein 90 inhibitor
HSP990	NVP-HSP990
lumi	luminespib; NVP-AUY922
pano	panobinostat
PCR	polymerase chain reaction
preinc	preincubation
