# Supplementary material for: Priming with HDAC Inhibitors Sensitizes Ovarian Cancer Cells to Treatment with Cisplatin and HSP90 Inhibitors
Source: Int J Mol Sci. 2020 Nov 5;21(21):8300. doi: 10.3390/ijms21218300 (PMC7663919; doi:10.3390/ijms21218300)
Supplement: Supplementary file 1 [file ijms-21-08300-s001.pdf]

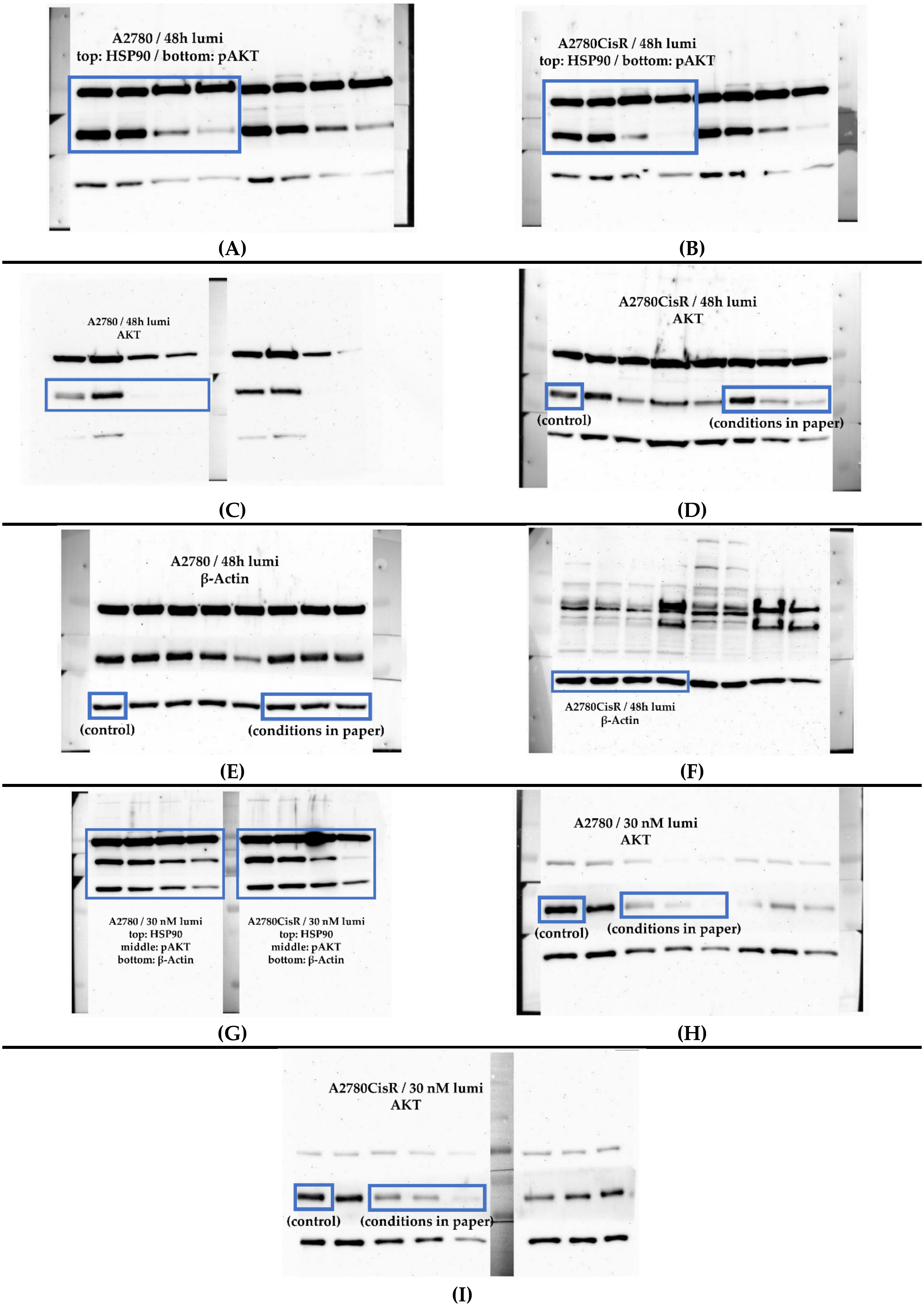

Figure S1. Effects of HSP90i and HDACi on cell growth and protein expression in A2780 and A2780CisR cells **(A-I).** Shown are the uncropped, labeled, and representative immunoblots from which Figure 1C was assembled.

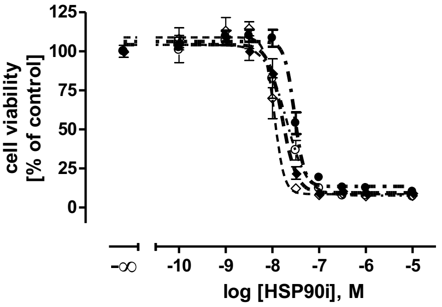

	IC_{50} [nM]
◇ A2780 NVP-AUY922	11.2
○ A2780 NVP-HSP990	20.1
◆ A2780CisR NVP-AUY922	16.4
● A2780CisR NVP-HSP990	29.8

(A)

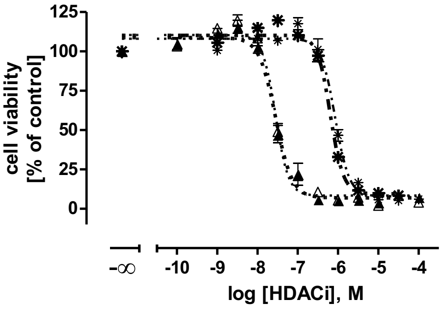

	IC_{50} [nM]
△ A2780 panobinostat	28.0
✳ A2780 LMK235	847
▲ A2780CisR panobinostat	28.2
✱ A2780CisR LMK235	644

(B)

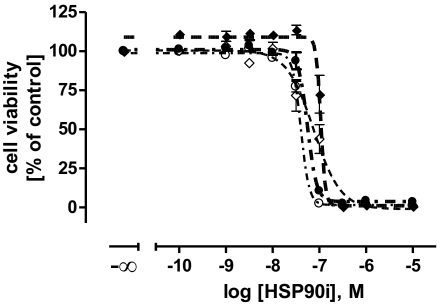

	IC_{50} [nM]
◇ CaOV3 NVP-AUY922	74.1
○ CaOV3 NVP-HSP990	40.9
◆ CaOV3CisR NVP-AUY922	108
● CaOV3CisR NVP-HSP990	55.6

(C)

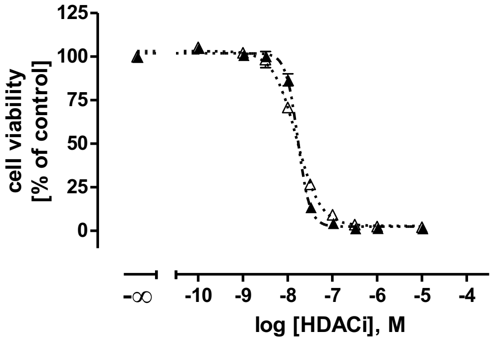

	IC_{50} [nM]
△ CaOV3 panobinostat	15.9
▲ CaOV3CisR panobinostat	16.9

(D)

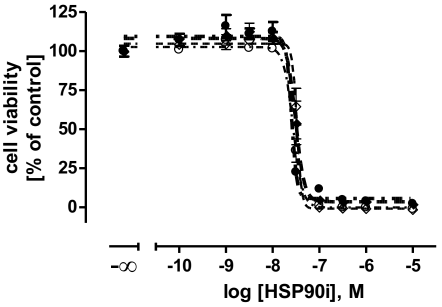

	IC_{50} [nM]
◇ OVCAR3 NVP-AUY922	33.5
○ OVCAR3 NVP-HSP990	27.6
◆ OVCAR3CisR NVP-AUY922	31.2
● OVCAR3CisR NVP-HSP990	25.0

(E)

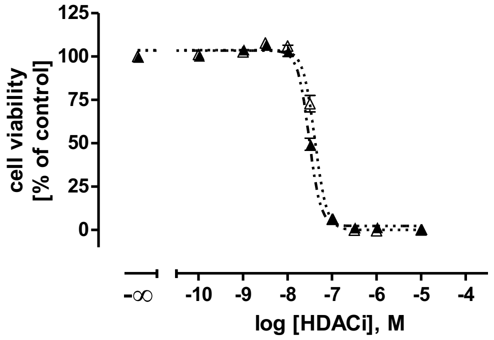

	IC_{50} [nM]
△ OVCAR3 panobinostat	41.3
▲ OVCAR3CisR panobinostat	30.5

(F)

Figure S2. Antiproliferative activity of HDACi and HSP90i against ovarian cancer cell lines. The cytotoxic activity of the HSP90i luminespib and NVP-HSP990 and the HDACi panobinostat was determined against A2780 (**A**,**B**), CaOV3 (**C**,**D**), OVCAR3 (**E**,**F**) and their cisplatin resistant sublines with a MTT assay after a 72 h incubation. The HDACi LMK235 was characterized at A2780 and A2780CisR (**A**,**B**). Data shown were determined in at least three independent experiments each performed in triplicate. Graphs show average ± SD. IC_{50}, pIC_{50}, and SEM are summarized in Table 1.

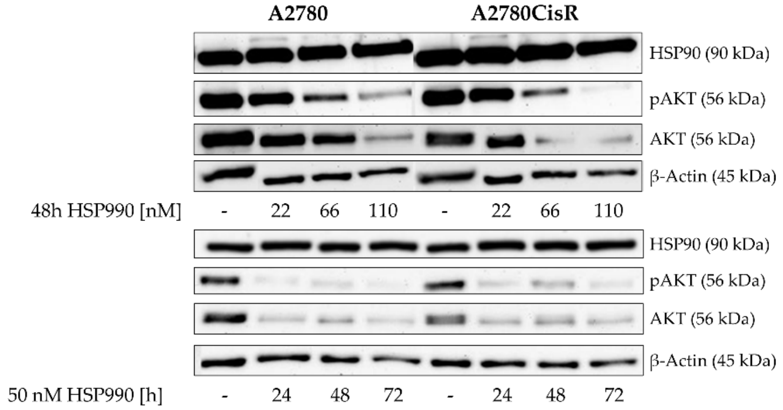

Figure S3. Concentration-dependent 48 h incubation with HSP990 (IC_{50}, three-fold and five-fold IC_{50}) and time-dependent incubation with three-fold IC_{50} of HSP990 led to a decrease in AKT expression and AKT phosphorylation. Shown is a representative experiment out of three.

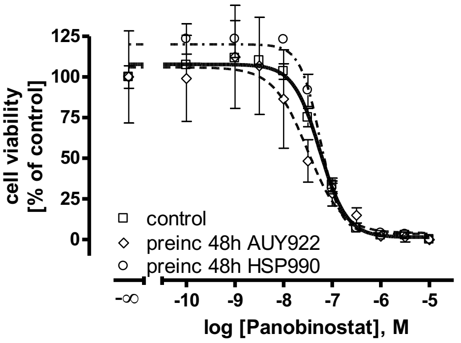

(A)

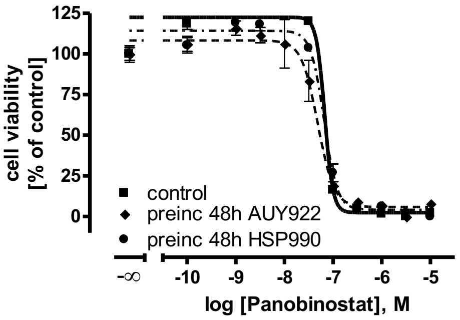

(B)

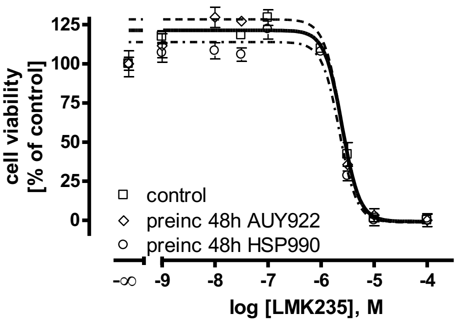

(C)

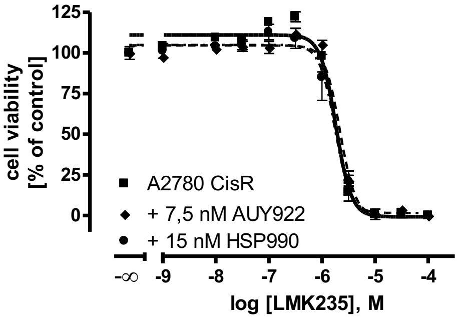

(D)

Figure S4. Influence on the cytotoxicity of HSP90i and HDACi on each other. A 48 h preincubation with the HSP90i (luminespib or HSP990 did not influence the cytotoxic activity of panobinostat (**A**,**B**) or LMK235 (**C**,**D**) in A2780 (**A**,**C**) and A2780CisR (**B**,**D**). Concentrations used were 5 nM luminespib and 10 nM NVP-HSP990 for A2780; 7.5 nM luminespib and 15 nM NVP-HSP990 for A2780CisR. Graphs shown are average ± SD from at least three independent experiments each performed in triplicate. Control is the concentration effect curve of cisplatin with the indicated cell line without any pretreatment. IC_{50} (pIC_{50} ± SEM) and significances are shown in Table S1.

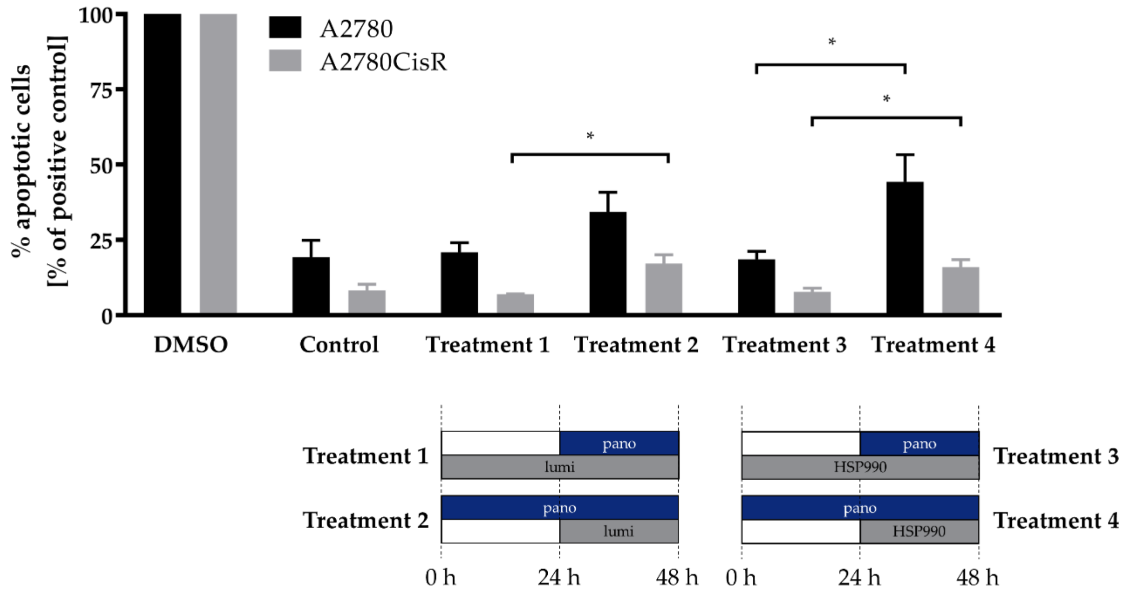

Figure S5. Apoptosis induction was analyzed in A2780 and A2780CisR after a 24 h incubation with an HSP90i or panobinostat followed by a 24 h incubation with both inhibitors in combination. Maximum incubation time did not exceed 48 h. Concentrations used were 10 nM panobinostat, 5 nM luminespib and 10 nM HSP990 in A2780 and 20 nM panobinostat, 7.5 luminespib and 15 nM HSP990 in A2780CisR. 10% DMSO for 24h was used as positive control for apoptosis induction. Values were normalized to the effect of 10% DMSO. Experimental treatment schemes 1–4: To analyze the effect of panobinostat (pano) on HSP90i (luminespib = lumi) and vice versa, one inhibitor was preincubated for 24 h before the other was applied for an additional 24 h incubation. Data shown are mean ± SEM of three independent experiments each carried out in triplicate. Statistical analysis was performed using t-test. Levels of significance: * ($p \leq 0.05$); ** ($p \leq 0.01$); *** ($p \leq 0.001$).

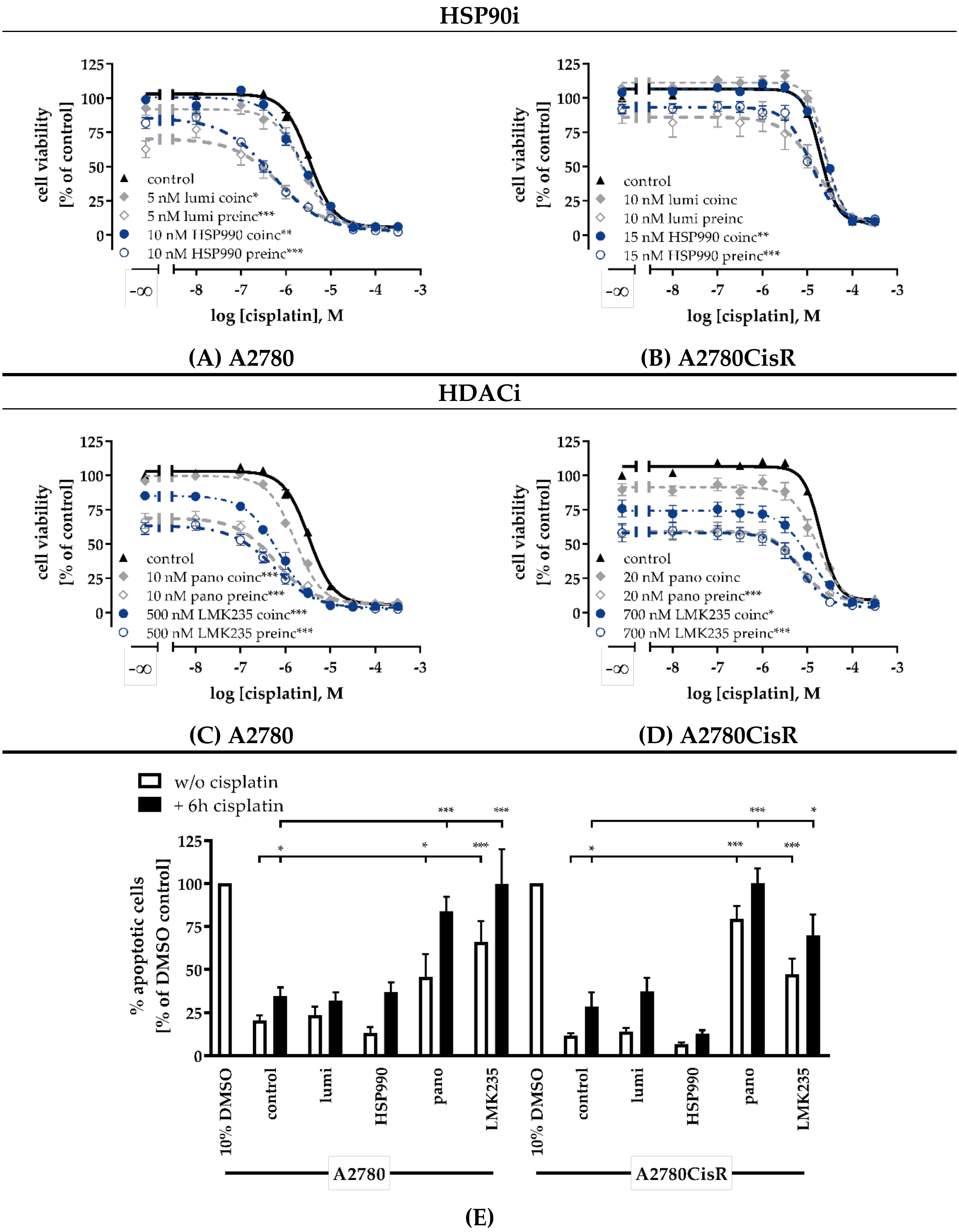

Figure S6. HSP90i and HDACi treatment enhance the activity of cisplatin in A2780 and A2780CisR. Coincubation of cisplatin (coinc) or a 48 h preincubation prior to cisplatin treatment (preinc) with HSP90i luminespib (lumi) or HSP990 (**A, B**) or HDACi panobinostat (pano) or LMK235 (**C, D**) enhanced the cytotoxic activity of cisplatin in A2780 and A2780CisR cells, respectively. (**E**) Apoptosis induction in A2780 and A2780CisR cells by HDACi or HSP90i with or without (w/o) cisplatin. HSP90i or HDACi were preincubated for 48 h prior to cisplatin treatment for 6h with an IC_{50} followed by 24 h recovery (without cisplatin). Concentrations used of HSP90i and HDACi were the same as denoted in (A–D). 10% DMSO was used as positive control for apoptosis induction. Data shown are mean ± SEM of three independent experiments. Corresponding IC_{50} values for A–D are shown in Table 2A. Statistical analysis was performed using t-test. Levels of significance: * ($p \leq 0.05$); ** ($p \leq 0.01$); *** ($p \leq 0.001$).

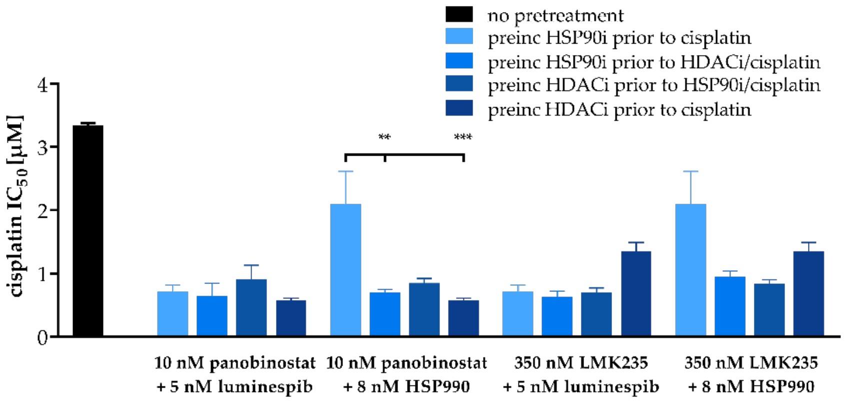

(A) A2780

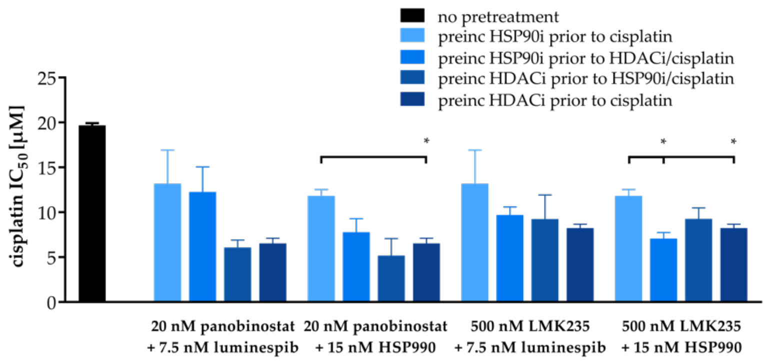

(B) A2780CisR

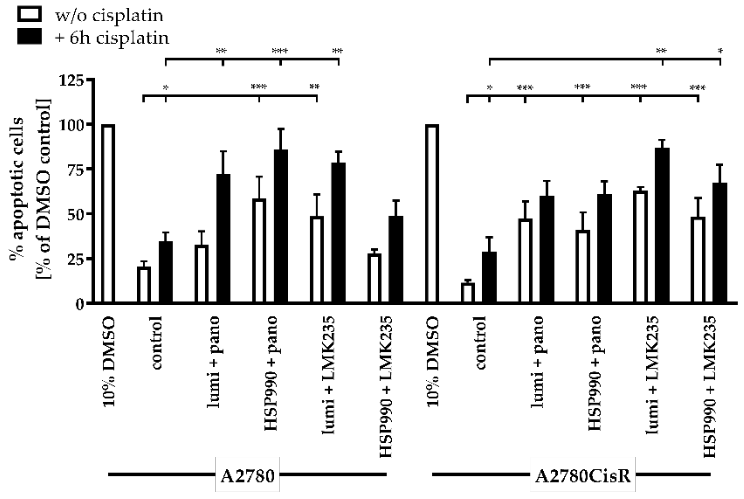

(C)

Figure S7. Triple combination treatment (HDACi, HSP90i, cisplatin) is not superior to dual combination of HDACi and cisplatin with regard to cisplatin cytotoxicity and apoptosis induction. 48 h preincubation of luminespib or HSP990 prior to addition of panobinostat or LMK235 plus cisplatin increased cisplatin sensitivity up to five-fold in A2780 (**A**) and A2780CisR (**B**). Cells were preincubated for 48 h with an HSP90i or HDACi prior to 72 h cisplatin plus HDACi or HSP90i. Corresponding results are also shown in Table 2B,C. (**C**) Apoptosis induction in A2780 and A2780CisR upon 48 h pretreatment with HDACi and/or HSP90i followed by 6 h cisplatin treatment (with an IC_{50} concentration) or solvent control (w/o cisplatin) and 24 h recovery without cisplatin. Concentrations used for apoptosis assay were the same as for cell viability assay (**A, B**) except for panobinostat in A2780CisR (25 nM). 10% DMSO was used as positive control for apoptosis induction. Data shown are mean ± SEM of three independent experiments. Statistical analysis was performed using t-test. Levels of significance: * ($p \le 0.05$); ** ($p \le 0.01$); *** ($p \le 0.001$).

Supplemental Method: Analyzing of cell cycle distribution. Cells were seeded in 6-well plates and treated with HSP90i or HDACi for 48 h. After treatment, cells were fixed and permeabilized in 70% ethanol at −20°C for at least 24 h. After washing cells with PBS, cells were stained with 1 µg/mL propidium iodide containing 0.1% Triton-X100 and 0.2 mg/mL DNAse-free RNAse A (AppliChem, Darmstadt, Germany). After 15 min incubation at 37 °C in the dark, cells were analyzed for DNA content by flow cytometry (Partec GmbH, Münster, Germany).

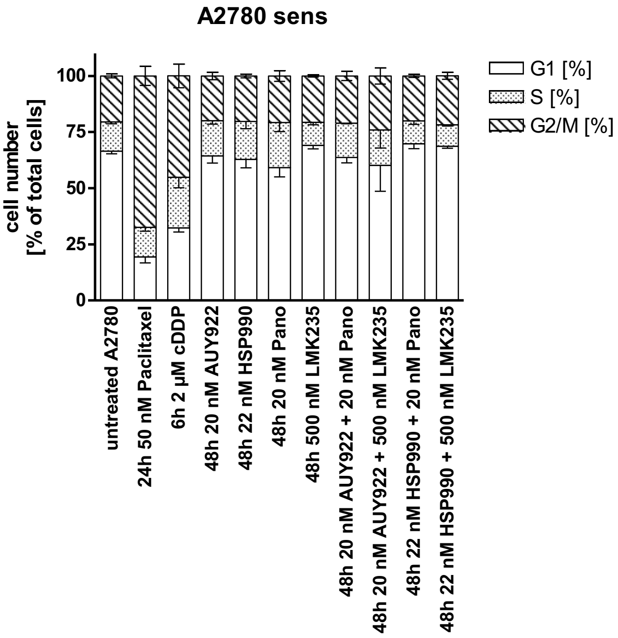

(A)

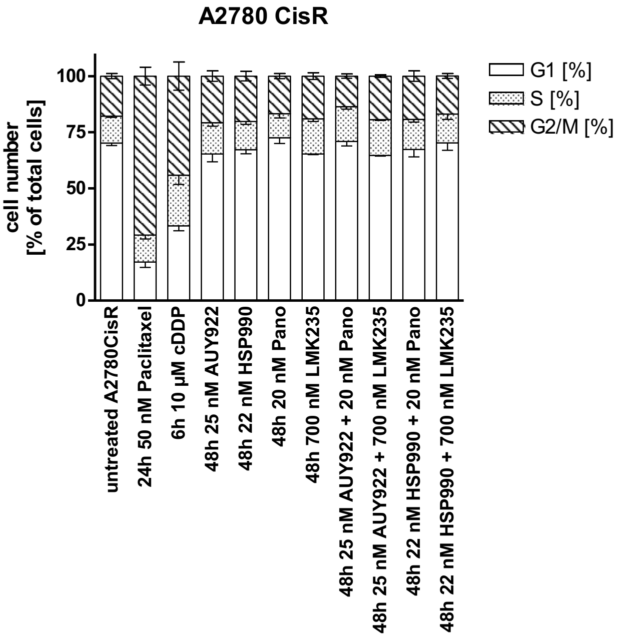

(B)

Figure S8. Effect of HDACi, HSP90i and drug combinations on cell cycle distribution of A2780 (**A**) and A2780CisR (**B**). Cells were incubated for 48 h with HDACi or HSP90i alone or in combination and stained with PI. For G2/M arrest control, 50 nM paclitaxel was incubated for 24 h. Graphs show average ± SEM from three independent experiments.

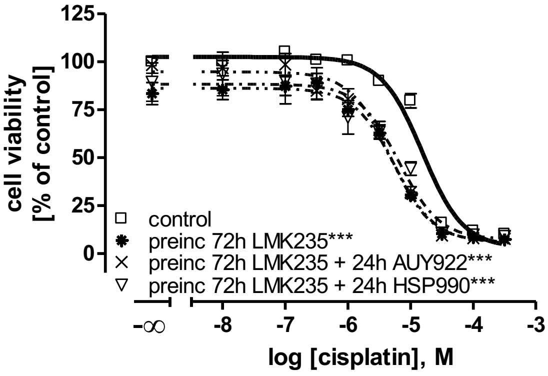

(A)

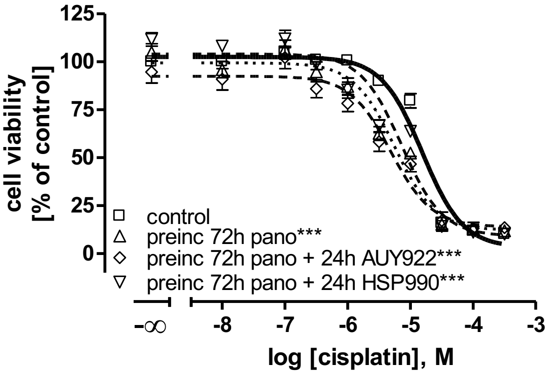

(B)

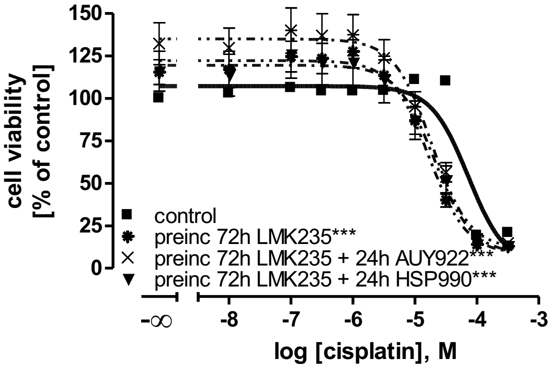

(C)

Figure S9. A 72 h preincubation with LMK235 (350 nM for A2780 and 500 nM for A2780CisR) (**A**,**B**) or panobinostat (10 nM for A2780 and 20 nM for A2780CisR) (**C**) or 48 h preincubation with HDACi followed by 24 h incubation together with HDACi and HSP90i increased cisplatin sensitivity after 48 h cisplatin treatment at A2780 (**C**) and A2780CisR (**A**,**B**) cells. Data shown are average ± SD from three independent experiments each carried out in triplicate. Control is the concentrations effect curve of cisplatin without any pretreatment. Statistical analysis was performed using t-test. Levels of significance: *** ($p \leq 0.001$).

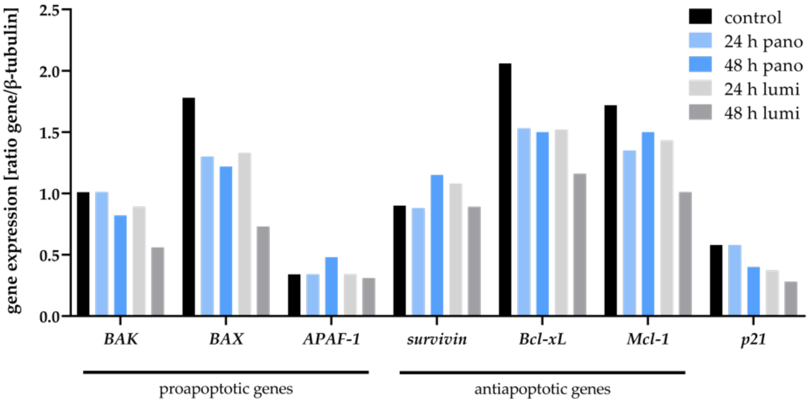

(A1) A2780

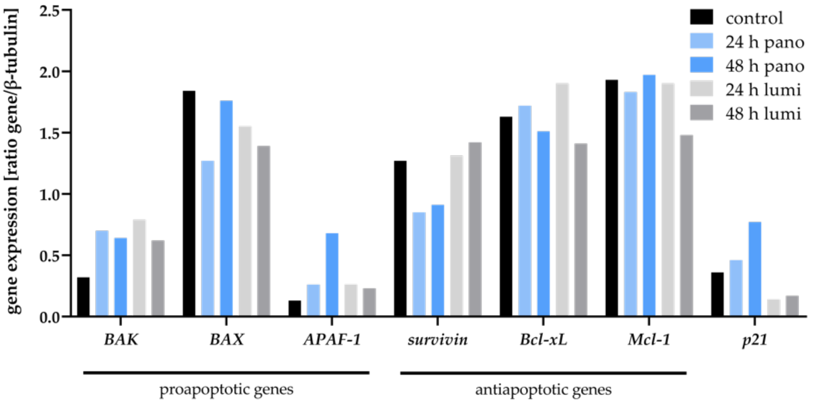

(A2) A2780CisR

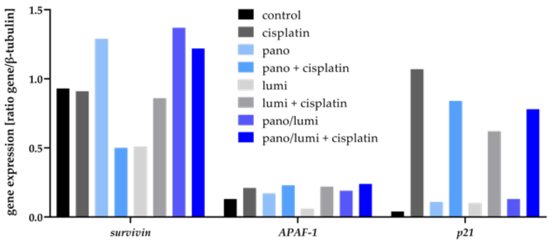

(B) A2780

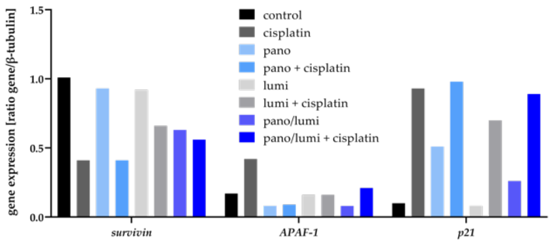

(C) A2780CisR

Figure S10. Effects of HDACi or HSP90i incubation or preincubation prior to cisplatin on apoptosis-related genes of A2780 **(A1,B)** and A2780CisR **(A2,C)** cells. Ratios of integrated densities of genes of interest and β-tubulin corresponding to PCR results shown in Figure 5.

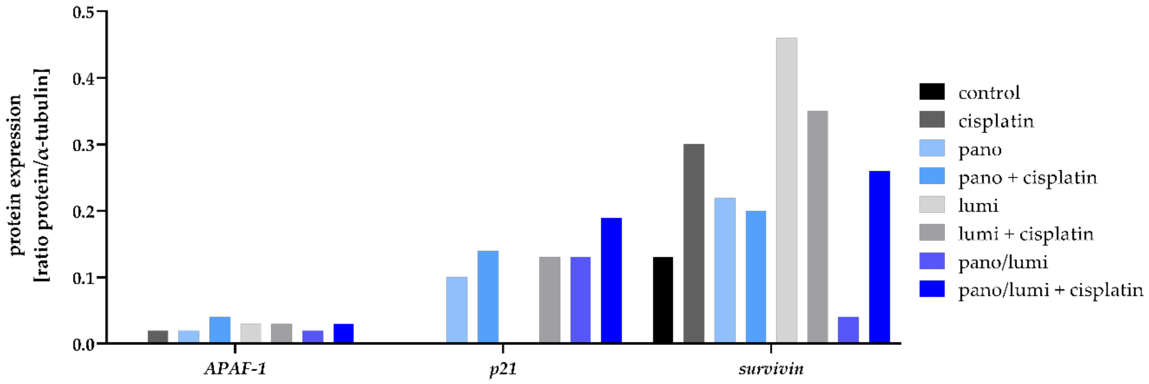

(A) A2780

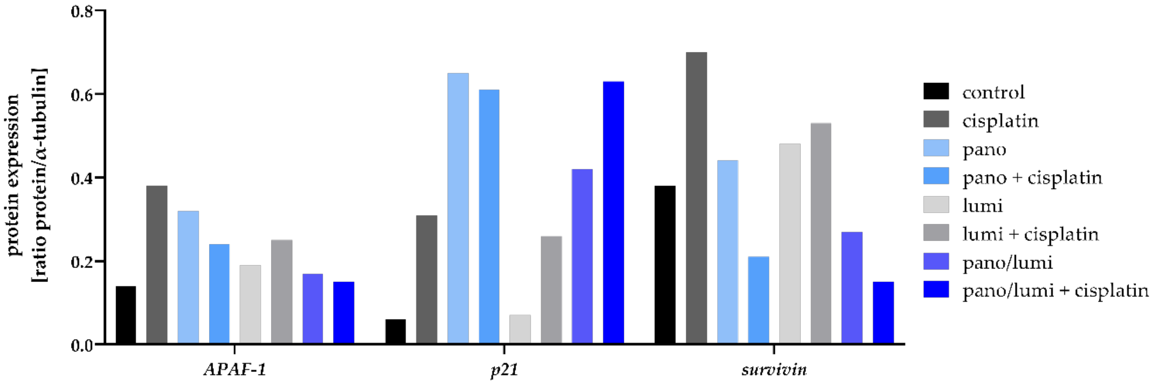

(B) A2780CisR

Figure S11. Effects of HDACi or HSP90i incubation or preincubation prior to cisplatin on protein expression levels of A2780 **(A)** and A2780CisR **(B)** cells. Ratios of integrated densities of genes of interest and α-tubulin corresponding to Western blot results shown in Figure 6.

Table S1. Influence on the cytotoxicity of HSP90i and HDACi on each other.

	**A2780**			
	**lumi**		**HSP990**	
	**IC_{50} (pIC_{50} ± SEM)**	**SF**	**IC_{50} (pIC_{50} ± SEM)**	**SF**
**control**	41.6 (7.38 ± 0.03)	-	74.0 (7.13 ± 0.04)	-
**pano preinc**	13.2 (7.88 ± 0.04)	3.2 ***	26.9 (7.57 ± 0.07)	2.8 ***
**LMK235 preinc**	29.1 (7.54 ± 0.02)	1.4 ***	40.8 (7.39 ± 0.05)	1.8 ***
	**pano**		**LMK235**	
**control**	56.2 (7.25 ± 0.03)	-	2,490 (5.60 ± 0.04)	-
**lumi preinc**	28.6 (7.54 ± 0.23)	2.0 (ns)	2,420 (5.62 ± 0.05)	1.0 (ns)
**HSP990 preinc**	58.8 (7.23 ± 0.07)	1.0 (ns)	2,240 (5.65 ± 0.04)	1.1 (ns)
**A2780CisR**				
	**lumi**		**HSP990**	
	**IC_{50} (pIC_{50} ± SEM)**	**SF**	**IC_{50} (pIC_{50} ± SEM)**	**SF**
**control**	44.2 (7.35 ± 0.03)	-	74.6 (7.13 ± 0.02)	-
**pano preinc**	13.6 (7.87 ± 0.10)	3.3 *	39.9 (7.40 ± 0.08)	1.9 **
**LMK235 preinc**	42.2 (7.38 ± 0.08)	1.0 (ns)	61.7 (7.21 ± 0.08)	1.2 (ns)
	**pano**		**LMK235**	
**control**	67.4 (7.17 ± 0.06)	-	1,830 (5.74 ± 0.04)	-
**lumi preinc**	48.6 (7.31 ± 0.05)	1.4 *	2,190 (5.66 ± 0.02)	0.8 (ns)
**HSP990 preinc**	65.5 (7.18 ± 0.04)	1.0 (ns)	1,770 (5.75 ± 0.05)	1.0 (ns)

IC_{50} values [nM] of MTT assays with HSP90i or HDACi incubation (72 h) at A2780 and A2780CisR after 48 h with indicated preincubation (preinc) of HDACi or HSP90i shown in Figures 2A–D and S3. Data shown are pooled data from three independent experiments. Statistical analysis was performed using t-test. Levels of significance: (ns) ($p > 0.05$); * ($p \leq 0.05$); ** ($p \leq 0.01$); *** ($p \leq 0.001$).

Table S2. Influence of dual or triple combinations with HDACi or HSP90i on the cytotoxic activity of cisplatin in A2780 and A2780CisR cells.

A – Dual Combination (inhibitor + cDDP)

cell line	cDDP (pIC_{50} ± SEM)								
	Control (cDDP only)	HSP90i				HDACi			
		luminespib		HSP990		panobinostat		LMK235	
		Coinc	preinc	coinc	preinc	coinc	preinc	coinc	preinc
A2780	5.48 ± 0.02	5.62 ± 0.06	6.15 ± 0.13	5.65 ± 0.05	6.30 ± 0.06	5.79 ± 0.03	6.24 ± 0.08	6.16 ± 0.04	6.25 ± 0.08
A2780CisR	4.71 ± 0.02	4.60 ± 0.03	4.88 ± 0.09	4.59 ± 0.02	4.93 ± 0.05	4.78 ± 0.07	5.19 ± 0.09	4.96 ± 0.09	5.15 ± 0.10

B – Triple Combination I (HSP90i prior to HSP90i + HDACi + cDDP)

		A2780 cDDP (pIC_{50} ± SEM)			A2780CisR cDDP (pIC_{50} ± SEM)		
		cDDP	HDACi/cDDP		cDDP	HDACi/cDDP	
			pano	LMK235		pano	LMK235
HSP90i	lumi	6.15 ± 0.13	6.19 ± 0.20	6.20 ± 0.11	4.88 ± 0.09	4.91 ± 0.18	5.01 ± 0.08
	HSP990	6.30 ± 0.06	6.16 ± 0.05	6.02 ± 0.07	4.93 ± 0.05	5.11 ± 0.14	5.15 ± 0.07

C – Triple Combination II (HDACi prior to HDACi + HSP90i + cDDP)

		A2780 cDDP (pIC_{50} ± SEM)			A2780CisR cDDP (pIC_{50} ± SEM)		
		cDDP	HSP90i/cDDP		cDDP	HSP90i/cDDP	
			lumi	HSP990		lumi	HSP990
HDACi	pano	5.79 ± 0.03	6.04 ± 0.16	6.07 ± 0.07	5.19 ± 0.09	5.22 ± 0.10	5.23 ± 0.11
	LMK235	6.25 ± 0.08	6.16 ± 0.08	6.08 ± 0.05	5.15 ± 0.10	5.04 ± 0.19	5.03 ± 0.11

(**A**) Dual combinations in A2780 and A2780CisR (data and concentrations from Figures S5,S6). (**B**) Triple combinations in A2780 and A2780CisR. Preincubation (preinc) means a 48 h preincubation with the indicated inhibitor followed by a 72 h incubation with cisplatin (**A**) and additionally if indicated with a HDACi/HSP90i (**C**).

Table S3. Shift factors of dual or triple combinations with HDACi or HSP90i on the cytotoxic activity of cisplatin in A2780 and A2780CisR cells.

A – Dual Combination (inhibitor + cDDP)

cell line	HSP90i				HDACi			
	luminespib		HSP990		panobinostat		LMK235	
	coinc	preinc	coinc	preinc	coinc	preinc	coinc	preinc
**A2780**	1.4 *	4.7 ***	1.5 **	6.7 ***	2.0 ***	5.9 ***	4.8 ***	5.9 ***
**A2780CisR**	0.8 *	1.5 ns	0.8 **	1.7 ***	1.2 ns	3.0 ***	1.8 *	2.8 ***

B – Triple Combination I (HSP90i prior to HSP90i + HDACi + cDDP)

		A2780			A2780CisR		
		cDDP	HDACi/cDDP		cDDP	HDACi/cDDP	
			pano	LMK235		pano	LMK235
**HSP90i**	**lumi**	4.7 ***	5.1 ***	5.3 ***	1.5 ns	1.6 ns	2.0 ***
	**HSP990**	6.7 ***	4.8 ***	3.5 ***	1.7 ***	2.5 *	2.8 ***

C – Triple Combination II (HDACi prior to HDACi + HSP90i + cDDP)

		A2780			A2780CisR		
		cDDP	HSP90i/cDDP		cDDP	HSP90i/cDDP	
			lumi	HSP990		lumi	HSP990
**HDACi**	**pano**	5.9 ***	3.7 ***	4.0 ***	3.0 ***	3.2 ***	3.3 *
	**LMK235**	5.9 ***	4.8 ***	4.0 ***	2.8 ***	2.1 *	2.1 **

Data shown are shift factors (SF) corresponding to drug combinations from Table 2. which were calculated as the ratio of IC_{50} of cisplatin and the IC_{50} of the corresponding drug combination. IC_{50} values are shown in Table 2, pIC_{50} and SEM are shown in Table S2. Statistical analysis was performed using t-test. Levels of significance: ns ($p > 0.05$); * ($p \le 0.05$); ** ($p \le 0.01$); *** ($p \le 0.001$).

Table S4. Influence of dual combinations with panobinostat or HSP990 on the cytotoxic activity of cisplatin in CaOV3, CaOV3CisR, OVCAR3, and OVCAR3CisR cells.

Cell Line	cDDP	+ 48 h pretreatment	
		HSP990	panobinostat
	pIC_{50} ± SEM	pIC_{50} ± SEM	pIC_{50} ± SEM
**CaOV3**	5.72 ± 0.01	6.10 ± 0.02	5.98 ± 0.05
**CaOV3CisR**	5.32 ± 0.02	5.50 ± 0.03	5.86 ± 0.15
**OVCAR3**	5.40 ± 0.02	5.56 ± 0.12	5.82 ± 0.09
**OVCAR3CisR**	4.42 ± 0.02	4.90 ± 0.24	5.10 ± 0.10

Data shown are pIC_{50} ± SEM from three independent experiments each carried out in triplicate. Dual combinations in CaOV3, OVCAR3, and their cisplatin resistant sublines. The concentrations used were 10 nM for panobinostat and 10 nM HSP990. IC_{50} values and SF are shown in Table 4.

Table S5. Effects of short-term treatment with HDACi or HSP90i on cisplatin sensitivity of A2780/A2780CisR ovarian cancer cells.

**A – A2780**			
	**IC_{50} (pIC_{50} ± SEM) [SF]**		
	**without inhibitor**	**+ 24 h 5 nM lumi**	**+ 24 h 8 nM HSP990**
**control (48 h cDDP)**	15.4 (4.81 ± 0.03)	-	-
**72 h 10 nM pano**	5.61 (5.25 ± 0.06) [2.7 ***]	4.88 (5.31 ± 0.08) [3.2 ***]	7.23 (5.14 ± 0.06) [2.1 ***]
**72 h 350 nM LMK235**	5.55 (5.26 ± 0.06) [2.8 ***]	4.78 (5.32 ± 0.06) [3.2 ***]	6.52 (5.19 ± 0.12) [2.4 ***]
**B – A2780CisR**			
	**IC_{50} (pIC_{50} ± SEM) [SF]**		
	**without inhibitor**	**+ 24 h 7.50 nM lumi**	**+ 24 h 15 nM HSP990**
**control (48 h cDDP)**	72.7 (4.14 ± 0.04)	-	-
**72 h 20 nM pano**	9.56 (5.02 ± 0.14) [7.6 ***]	11.7 (4.93 ± 0.23) [6.2 ***]	9.88 (5.01 ± 0.27) [7.4 ***]
**72 h 500 nM LMK235**	17.0 (4.77 ± 0.09) [4.3 ***]	18.7 (4.73 ± 0.10) [3.9 ***]	20.5 (4.69 ± 0.13) [3.5 ***]

Data shown are IC_{50} values in µM and pIC_{50} ± SEM of cisplatin (48 h incubation) determined by MTT assays against A2780 (**A**) and A2780CisR (**B**) with inhibitor incubation. Data were obtained from three independent experiments each carried out in triplicate. Shift factors (SF) were calculated as the ratio of IC_{50} of cisplatin and the IC_{50} of the corresponding drug combination. Lumi means luminespib and pano means panobinostat. Concentrations used as described in Figure 8. Corresponding Curves are shown in Figures 4A and S8. Statistical analysis was performed using t-test. Levels of significance: *** ($p \leq 0.001$).

Table S6. Influence of HDACi/HSP90i preincubation on cisplatin sensitivity of non-tumor cell line HEK293.

preincubation condition	IC_{50} (pIC_{50} ± SEM)	SF
**control**	3.09 (5.51 ± 0.02)	-
**2 nM luminespib**	4.47 (5.35 ± 0.03)	0.7
**5 nM HSP990**	2.60 (5.59 ± 0.05)	1.2
**10 nM panobinostat**	2.54 (5.60 ± 0.07)	1.2
**350 nM LMK235**	1.85 (5.73 ± 0.07)	1.7

Data shown are IC_{50} values in µM and pIC_{50} ± SEM of cisplatin after a 72 h incubation determined with MTT assays against HEK293 cells. HEK293 cells were preincubated with the indicated inhibitors for 48 h prior to cisplatin administration. Shift factors (SF) were calculated as the ratio of IC_{50} of cisplatin ("control") and the IC_{50} of the corresponding drug combination. Graphs are shown in Figure 7. Data were obtained from three independent experiments each carried out in triplicate. The shift factors shown were not significantly different from control. Statistical analysis was performed using t-test.

Table S7. Results of STR analysis of A2780 and A2780CisR.

Loci	A2780	A2780 (ECACC)	A2780CisR	A2780CisR (ECACC)
**D5S818**	11, 12	11, 12	11, 12	11
**D16S539**	11, 13	11, 13	11, 13	11, 13
**vWA**	15, 16	15, 16	15, 16	15, 16
**D13S317**	12, 13	12, 13	13	13
**CSF1PO**	10, 11	10, 11	10, 11	10, 11
**TPOX**	8, 10	8, 10	8	8, 10
**TH01**	6	6	6	6
**D21S11**	28	-	28	-
**D7S820**	10	10	10	10
**AMEL**	X	X	X	X

Shown are the results of the short tandem repeat (STR) analysis of the cell lines A2780 and A2780CisR. The results were compared with the data of the cell bank ECACC and it can be stated that A2780 and A2780CisR were successfully authenticated.
